# Identification of Unequally Represented Founder Viruses Among Tissues in Very Early SIV Rectal Transmission

**DOI:** 10.3389/fmicb.2018.00557

**Published:** 2018-03-29

**Authors:** Jian Chen, Yanqin Ren, Lance Daharsh, Lu Liu, Guobin Kang, Qingsheng Li, Qiang Wei, Yanmin Wan, Jianqing Xu

**Affiliations:** ^1^Key Laboratory of Medical Molecular Virology of Ministry of Education/Health, Scientific Research Center, Shanghai Public Health Clinical Center & Institutes of Biomedical Sciences, Shanghai Medical College, Fudan University, Shanghai, China; ^2^Department of Microbiology, Immunology, and Tropical Medicine, George Washington University School of Medicine and Health Sciences, Washington, DC, United States; ^3^Nebraska Center for Virology, School of Biological Sciences, University of Nebraska-Lincoln, Lincoln, NE, United States; ^4^Key Laboratory of Human Disease Comparative Medicine, Chinese Ministry of Health, Beijing Key Laboratory for Animal Models of Emerging and Remerging Infectious Diseases, Institute of Laboratory Animal Science, Chinese Academy of Medical Sciences and Comparative Medicine Center, Peking Union Medical College, Beijing, China

**Keywords:** Chinese rhesus macaque, rectal transmission, SIV, transmitted/founder virus, very early virological events, CG dinucleotide, zinc finger antiviral protein

## Abstract

Characterizing the transmitted/founder (T/F) viruses of multi-variant SIV infection may shed new light on the understanding of mucosal transmission. We intrarectally inoculated six Chinese rhesus macaques with a single high dose of SIVmac251 (3.1 × 10^4^ TCID_50_) and obtained 985 full-length *env* sequences from multiple tissues at 6 and 10 days post-infection by single genome amplification (SGA). All 6 monkeys were infected with a range of 2 to 8 T/F viruses and the dominant variants from the inoculum were still dominant in different tissues from each monkey. Interestingly, our data showed that a cluster of rare T/F viruses was unequally represented in different tissues. This cluster of rare T/F viruses phylogenetically related to the non-dominant SIV variants in the inoculum and was not detected in any rectum tissues, but could be identified in the descending colon, jejunum, spleen, or plasma. In 2 out of 6 macaques, identical SIVmac251 variants belonging to this cluster were detected simultaneously in descending colon/jejunum and the inoculum. We also demonstrated that the average CG dinucleotide frequency of these rare T/F viruses found in tissues, as well as non-dominant variants in the inoculum, was significantly higher than the dominant T/F viruses in tissues and the inoculum. Collectively, these findings suggest that descending colon/jejunum might be more susceptible than rectum to SIV in the very early phase of infection. And host CG suppression, which was previously shown to inhibit HIV replication *in vitro*, may also contribute to the bottleneck selection during *in vivo* transmission.

## Introduction

Characterizing the T/F viruses during mucosal infection is important for HIV-1 vaccine and transmission studies (Shaw and Hunter, 2012). By investigating quasispecies complexity in HIV-1 infected individuals, early studies have suggested that genetically diverse viral quasispecies in chronic infections were presumably resulting from one or few closely related T/F viruses (Wolfs et al., 1992; Wolinsky et al., 1992; Zhang et al., 1993; Zhu et al., 1993, 1996; Poss et al., 1995; Long et al., 2000; Learn et al., 2002; Derdeyn et al., 2004; Grobler et al., 2004; Ritola et al., 2004; Sagar et al., 2004, 2009). Based on these observations, Keele and colleagues characterized T/F viruses more precisely using single-genome amplification (SGA) and showed that only a limited number of virus variants are transmitted through mucosal transmission in both human and non-human primate (Keele et al., 2008, 2009) which is widely believed to be the consequence of “transmission bottleneck” selection (Kariuki et al., 2017). Although similar percentages of heterosexual and MSM transmissions can be traced back to a single founder virus (Keele et al., 2009), several lines of evidences have suggested that the risk of rectal transmission is relatively higher (Lane et al., 2006; Kalichman et al., 2009; Veldhuijzen et al., 2011) and the multiplicity of rectal HIV-1 infection was also greater than genital tract transmission (Li et al., 2010). These differences suggest that it could be more difficult to prevent rectal HIV transmission than to prevent vaginal transmission. More evidence has been provided in non-human primate studies on topically applied ARVs that showed better protection against vaginal transmission (Parikh et al., 2009) than rectal transmission (Cranage et al., 2008).

The less stringent bottleneck of rectal transmission may be attributed to anatomical and histological differences between the genitourinary tracts and the lower intestine (Li et al., 2010). However, the exact mechanisms are still elusive. An intriguing study done in rhesus macaques intrarectally infected with a single high dose of SIV_mac251_ showed a higher number of transmitted virus variants as a result of increased inoculum dosage (Liu et al., 2010), further indicating that the rectal mucosa may be more vulnerable to HIV infection than previously thought (Keele et al., 2009). To understand the nature of the “transmission bottleneck,” intensive efforts have been put into identifying common features of HIV-1 T/F viruses (Joseph et al., 2015), however, no consistent signature has been found in human studies except that most T/F viruses use the CCR5 co-receptor (Keele et al., 2008; Ping et al., 2013).

A major advantage of using the SIV infected rhesus macaque model to investigate T/F viruses is that the exact genome sequences of inoculating viruses can be obtained, which makes comparisons between the transmitted and non-transmitted variants possible. Previously, most non-human primate transmission studies have focused only on comparing inoculum viruses with viruses isolated from plasma (Keele et al., 2009; Liu et al., 2010), leaving T/F virus variants within the intestinal mucosa largely unknown. Recently the T/F virus distribution across anal-rectum, peripheral blood, and distant lymph nodes at the very early phase of SIVmac251 rectal transmission in Indian rhesus macaques was characterized (Yuan et al., 2017). However, this study did not investigate the transmitted viruses in other parts of intestinal mucosa, e.g., the descending colon, which could also be potential portals of entry (Smedley et al., 2014). Considering the dramatic anatomical and immunohistological variation across different parts of the intestinal mucosa, we speculated that characterizing T/F viruses across multiple sites is of high importance for understanding the HIV rectal transmission bottleneck.

## Materials and methods

### Ethics statement

This study was approved by the Institutional Animal Care and Use Committee (IACUC) at the Institute of Laboratory Animal Science, Chinese Academy of Medical Sciences (ILAS, CAMS). All animal experimental procedures were performed in an Animal Bio-Safety Level 3 (ABSL-3) laboratory, which is fully accredited by the Association for Assessment and Accreditation of Laboratory Animal Care (AAALAC), International. This study was carried out in strict compliance with the “Guide for the Care and Use of Laboratory Animals of the Institute of Laboratory Animal Science (est. 2006)” and “The use of non-human primates in research of the Institute of Laboratory Animal Science (est. 2006)” to ensure personnel safety and animal welfare. All the Chinese rhesus macaques enrolled this study were negative for HIV-2, SIV, type-D retrovirus, and simian T cell lymphotropic virus-1 when the study was initiated.

### Experimental design and tissue collection

**Figure 1:**
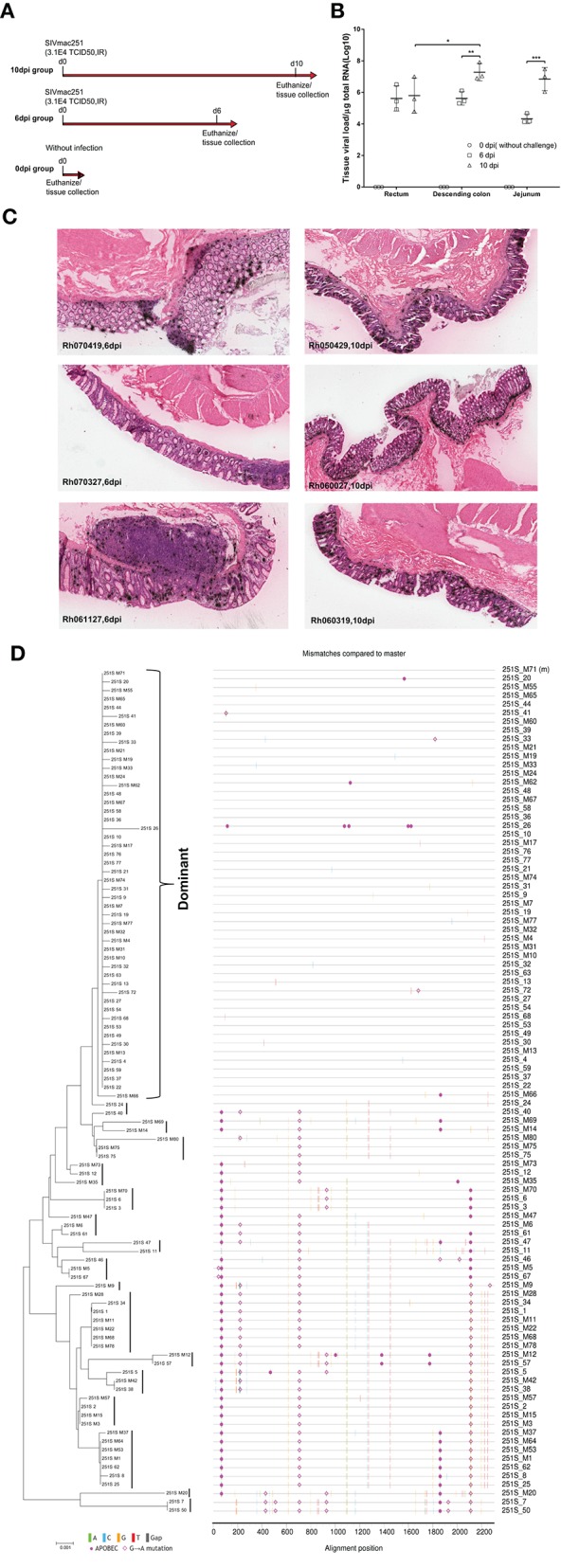
Experimental design and the genetic make-up of SIVmac251 inoculum. **(A)** Three groups of Chinese rhesus macaques (3 monkeys/group) were enrolled in this study. Two groups were intrarectally infected with a single high dose of SIVmac251 (3.1 × 10^4^ TCID_50_/monkey) and euthanized at 6 or 10 dpi. **(B**,**C)** Tissue viral RNA loads were measured by quantitative PCR **(B)** and virus replication in rectal mucosa was detected by *in situ* hybridization **(C)**. **(D)** The inoculum stocks of SIVmac251 were evaluated for *env* diversity by NJ phylogeny and Highlighter plots. Reading frame intact *env* sequences were shown in parallel in NJ phylogeny and Highlighter plots. Nucleotide polymorphisms in the highlighter plot are indicated by a colored mark. Thymine is represented in red, guanine in orange, adenine in green, cytosine in blue, pink filled circles denote APOBEC signatures, open diamonds represent G-to-A conversions, and gaps are shown in gray in the highlighter plots. Bar length indicates 0.001 nucleotide substitutions per site. ^*^P < 0.05; ^**^P < 0.01; ^***^P < 0.001.

Nine male adult rhesus macaques (*Macaca mulatta*) of Chinese origin were randomly divided into 3 equal groups and housed at the institute of laboratory animal science, Chinese Academy of Medical Sciences (ILAS, CAMS). Six animals were intrarectally inoculated with 1 ml (3.1 × 10^4^ TCID_50_) cell-free SIVmac251 and euthanized at 6 dpi (*n* = 3, Rh061127, Rh070327, Rh070419) or 10 dpi (*n* = 3, Rh060027, Rh060319, Rh050429). The SIV_mac251_ virus stock, kindly provided by Dr. Qiang Wei (ILAS, CAMS), is CCR5-tropic and was expanded in rhesus macaque peripheral blood mononuclear cells (PBMCs) (Cong et al., 2015) and titrated using a rhesus macaque PBMC based assay (Marthas et al., 2001). Another three monkeys were used as non-infected controls and sacrificed on day 0 (Figure 1A). After euthanasia, plasma, spleen, anal rectum, descending colon, and jejunum were collected and frozen at −80°C until use.

### Viral RNA extraction and cDNA synthesis

Plasma and virus stock were thawed on ice and viral RNA was isolated using the QIAamp viral RNA mini kit (# 52904, Qiagen, Valencia, CA, USA) according to the manufacturer's protocol. Total RNA was extracted from homogenized tissues using RNAzol (# 190, MRC, Cincinnati, OH, USA) and purified using RNeasy Mini Kit (# 74106, Qiagen). RNA quantity and quality was measured by Nanodrop (Thermo Scientific, Waltham, MA USA). One microgram of tissue or viral RNA was transcribed into cDNA by using random primers and the Moloney murine leukemia virus (M-MLV) reverse transcriptase (#M1302-40KU, Promega, Charbonnieres, France). cDNA was stored at −20°C until use.

### Viral load detection

Plasma and the tissue levels of SIV RNA were determined by following previously published methods (Cline et al., 2005; Ren et al., 2016). All samples were repeatedly quantified in two independent experiments.

### Single genome amplification and sanger sequencing

Near full-length 2.3 kb SIVmac251 *env* was amplified using nested PCR by following previously published protocols (Stone et al., 2010). Briefly, cDNA was serially diluted to obtain less than 30% positivity in the total PCR reactions. At this dilution, most positive wells contain amplicons derived from a single cDNA molecule. This was confirmed in every positive reaction by inspection of the sequence for double peaks after sanger sequencing. Any sequence with evidence of mixed bases was excluded from further analysis. PCR amplification was performed in a 20 μl reaction. The first-round of PCR was performed with forward primer 10 μM 251envF1 (5′-CAG TCT TTT ATG GTG TAC CAG CTT GGA GGA ATG-3′), and reverse primer 10 μM 251envR1 (5′-GAG GAT CCA TCT TCC ACC TCT CCT AAG AGT C-3′), which generated an ~2.5 kb product. PCR was performed in 96-well PCR plates under the following parameters: 94°C for 2 min, 35 cycles of 94°C for 20 s, 56°C for 30 s, and 72°C for 2.5 min, and an extension step of 72°C for 10 min. The second-round PCR was conducted using the same condition as the first-round PCR except using 2 μl of the first round PCR products as template and running 40 cycles with forward primer 10 μM 251envF2 (5′-GGA ACA ACT CAG TGC CTA CCA GAT AAT GGT G-3′), and reverse primer 10 μM 251envR2 (5′-GTA GGT CAG TTC AGT CCT GAG GAC TTC TCG-3′). PCR products were separated by 1% agarose gel electrophoresis and the positive bands were excised from the gels and purified using the QIAquick Gel Extraction Kit (#28706, Qiagen, Valencia, CA, USA). Purified PCR products were sequenced with six overlapping primers using Sanger method at Biosune Biotechnology (Shanghai, China). Sequencing primers are listed as following.

C41950 primer F: 5′-GGAACAACTCAGTGCCTACCAGATAAT-3′,

C41951primer R: 5′-GTAG GTCAGTTCAGTCCTGAGGACTTC-3′,

C42016 1F.W1F: 5′-TGCACAAGGATGATGG AGAC-3′,

C42018 1R.W1F: 5′-GTACTTCTCGATGGCAGTGA-3′,

C42053 1F.W1Cnew: 5′-CT CTTGTTCCAAGCCTGTGC-3′,

C42054 1R.W1Cnew: 5′-GGTATAGGCCAGTGTTCTCT-3′.

### SIVmac251 viral RNA detection in tissues using ISH

*In situ* hybridization (ISH) was conducted according to previously published methods (Li et al., 2005; Destache et al., 2016). Briefly, animal intestinal tissues were collected after euthanasia and were fixed in 4% paraformaldehyde. Approximately 6 μm tissue sections were cut and adhered to a SuperFrost plus slide, fixed, and air dried. The sections were then rehydrated, permeabilized, and acetylated prior to hybridization to 35S-labeled SIV riboprobes. After washing and digestion with RNase, sections were coated with nuclear track emulsion, exposed for 7 days, developed, and counterstained with hematoxylin and eosin (H&E) stain.

### Sequence analysis

To ensure that the *env* sequence was derived from a single genome, chromatograms of sanger sequencing were first manually examined for overlaid multiple peaks, which indicated the presence of PCR generated recombination events, Taq polymerase errors, or multiple variant templates. Sequences with overlaid multiple peaks were excluded from further analysis. Next, the sequences were aligned by using ClustalW and only the open reading frame(ORF) intact sequences were selected for downstream phylogenetic analysis. Neighbor-joining phylogenetic trees were constructed using MEGA7 and highlighter diagrams were generated by the online highlighter tool (www.hiv.lanl.gov). The number of T/F lineages was counted by inspecting neighbor-joining phylogenies and the Highlighter plots as being described in previous studies (Keele et al., 2008, 2009; Stone et al., 2010). While identifying a T/F variant lineage, the following criteria were applied: First, branches containing two or more identical sequences; Second, sequences that closely clustered on the same branch; third, individual sequences showing recombination signature were excluded from counting. Sequence divergence was analyzed using MEGA7. To analyze CG dinucleotide frequency, sequence files in fasta format were read by a perl script in order to count single nucleotide and dinucleotide frequencies within the sequence. The perl script was adapted from the Biostars bioinformatics forum user biolab. Sequences analyzed in this study were uploaded to Genbank (SRA accession: SRP133347).

### Statistical analysis

**Figure 2:**
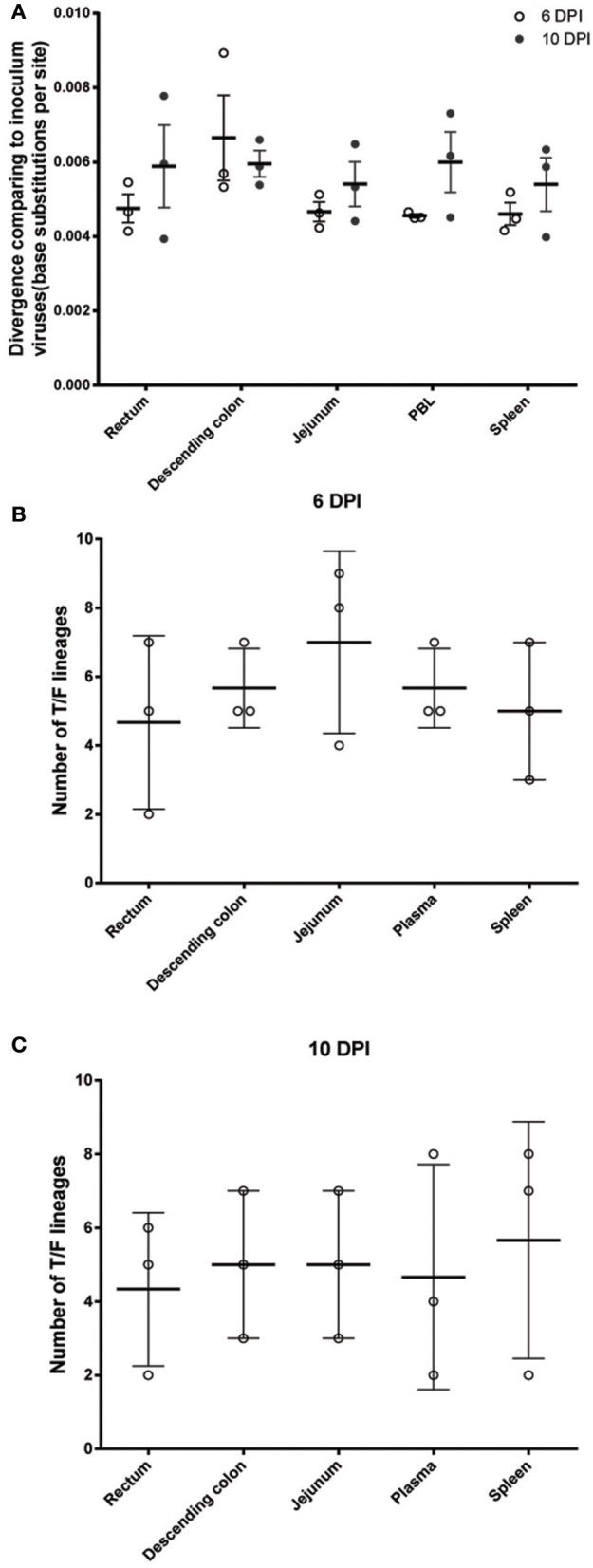
Divergence and diversity analyses of transmitted/founder viruses isolated from different tissue compartments **(A)**. The numbers of base substitutions per site from averaged over all sequence pairs between the inoculum and each tissue compartment (divergence from inoculum, calculated using MEGA7). The number of transmitted variants varied among different tissue compartments at both 6 dpi **(B)** and 10 dpi **(C)**. Although no significant difference was observed, the number of T/F lineages in rectum tended to be lower than other tissue compartments. Moreover, the numbers of virus lineages in tissues were all less than the inoculum, which contained at least 17 lineages.

Data are presented as mean ± *SD* in Figures 1B, 2, **5B**. Comparisons among multiple groups were conducted by the method of ordinary one-way ANOVA and each group was compared to all other groups by Tukey`s multiple comparisons (GraphPad Software, Inc., San Diego, California, USA).

## Results

### The SIV_mac251_ inoculum contained diverse variants and all monkeys were successfully infected after a single anorectal exposure

In total, nine Chinese rhesus macaques were enrolled in this study. Six were intrarectally inoculated with 3.1 × 10^4^ TCID_50_ cell-free SIV_mac251_ and were euthanized at either 6 or 10 days post-infection (dpi) (Figure 1A). To characterize the diversity of virus in the inoculum, a total of 98 ORF intact *env* sequences were generated by SGA and sanger sequencing. We found that the inoculum contained at least 17 different lineages and the dominant one comprised 47 nearly identical sequences (Figure 1D). The dominant lineage percentage in the inoculum was 48%, which was comparable to a recent report (44%) (Yuan et al., 2017). The overall nucleotide diversity among this virus stock was 0.005 base substitution per site, which was slightly lower than previously published data (0.008 base substitution per site) (Keele et al., 2009). To confirm the establishment of infection, viral RNA in intestinal tissues was detected by qPCR (Figure 1B) and *in situ* hybridization (Figure 1C and Figure S9). Both assays confirmed that all monkeys in the 6 and 10 dpi groups were successfully infected. Viral loads in jejunum and descending colon significantly increased from 6 to 10 dpi, while viral loads remained stable in rectum tissues (Figure 1B). Additionally, at 10 dpi the average viral load in the descending colon was significantly higher than that in the rectum (Figure 1B).

### Rectum was not the anatomical site that contained the most diverse T/F viruses at both 6 and 10 dpi

**Figure 3:**
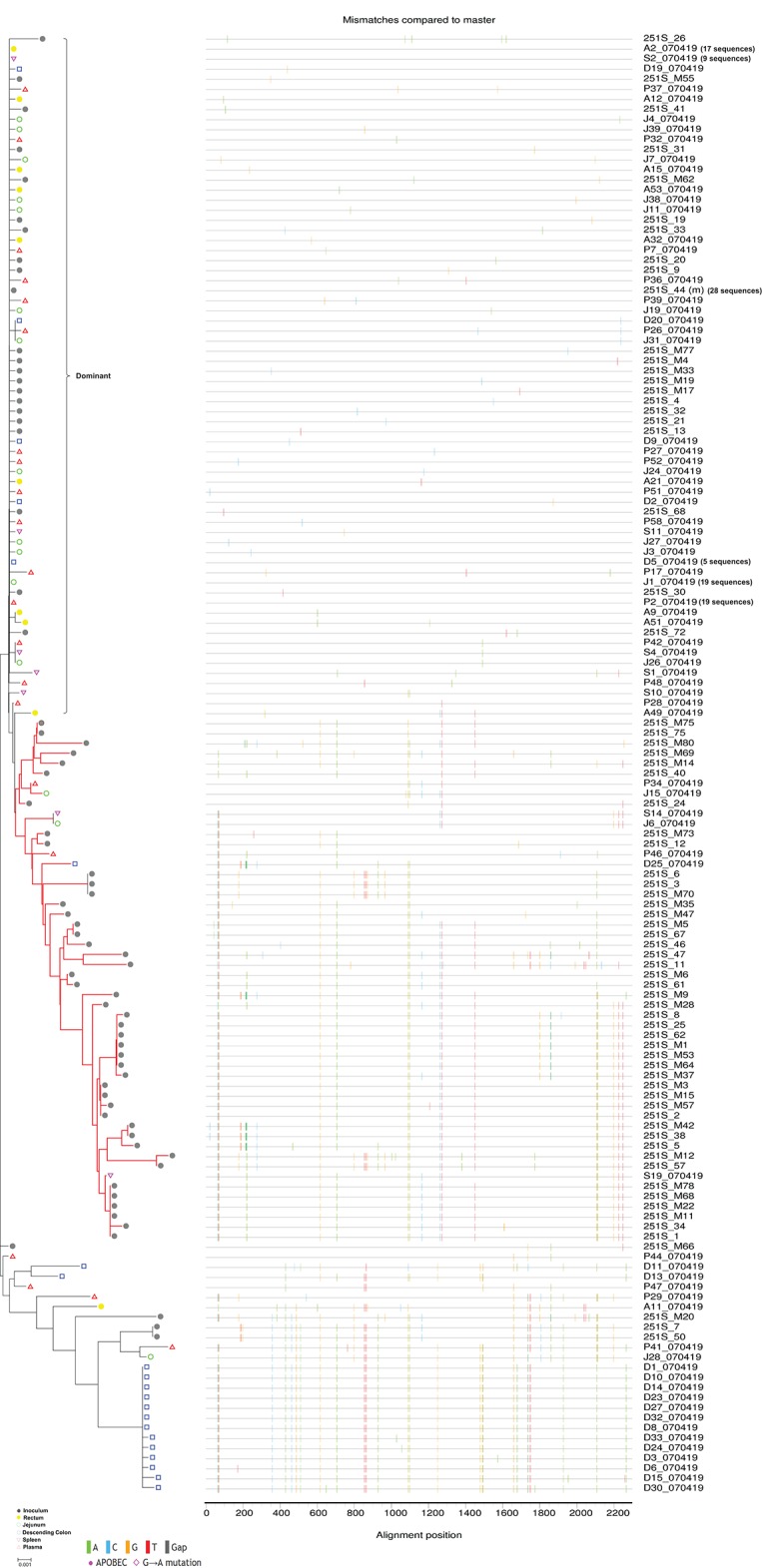
N-J tree and Highlighter plots of SGA-derived *env* nucleotide sequences from 6 dpi macaque Rh070419. Sequences derived from different tissue compartments are shown in N-J phylogeny and Highlighter plots. In the phylogeny plot, the inoculum viruses are depicted in closed gray circles, rectum viruses in closed yellow circles, jejunum viruses in green circles, descending colon viruses in blue squares, spleen viruses in purple downward triangles, and peripheral blood viruses in red upward triangles. The red branches highlight a cluster of variants that was absent from rectum. The right bracket indicates the dominant variants. Identical sequences in the dominant cluster are represented by only one sequence for each tissue compartment. Actual number of sequences is shown in the bracket following the seq ID. Bar length represents 0.001 nucleotide substitutions per site.

**Figure 4:**
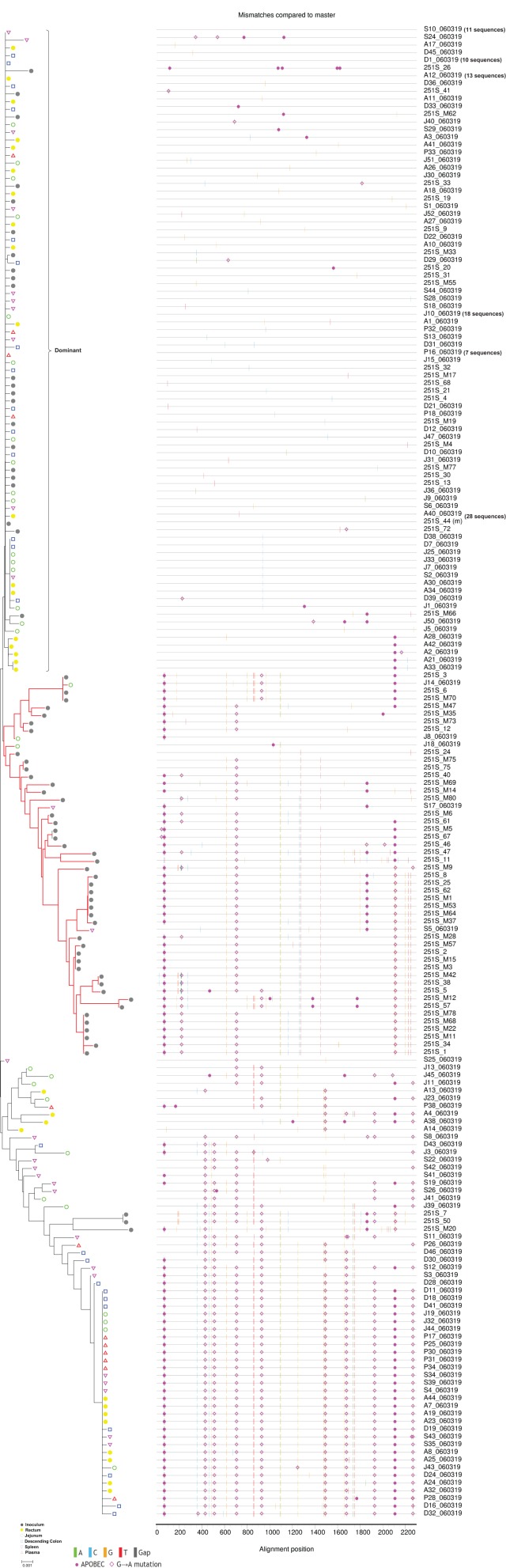
N-J tree and Highlighter plots of SGA-derived *env* nucleotide sequences from 10 dpi macaque Rh060319. Sequences derived from different tissue compartments are shown in N-J phylogeny and Highlighter plots. In the phylogeny plot, the inoculum viruses are depicted in closed gray circles, rectum viruses in closed yellow circles, jejunum viruses in green circles, descending colon viruses in blue squares, spleen viruses in purple downward triangles, and peripheral blood viruses in red upward triangles. The red branches highlight a cluster of variants that was absent from rectum. The right bracket indicates the dominant variants. Identical sequences in the dominant cluster are represented by only one sequence for each tissue compartment. Actual number of sequences is shown in the bracket following the seq ID. Bar length represents 0.001 nucleotide substitutions per site.

To delineate the anatomical distribution of the T/F viruses, a total of 985 ORF intact *env* sequences were generated from rectum, descending colon, jejunum, peripheral blood, and spleen of each monkey (Table 1). Interestingly, despite being the site of inoculation, our data showed that rectum did not contain the most diverse transmitted viruses. The average divergence (compared to the inoculum) tended to be higher in the descending colon, compared to other tissue compartments (Figure 2A), although statistical significance was not reached. We further enumerated the number of T/F viruses in the different tissue compartments of each monkey (Figures S1–S6) and found that the number of T/F viruses tended to be lower in rectum at both 6 and 10 dpi (Figures 2B,C). For each tissue compartment, the number of T/F viruses remained relatively stable between 6 and 10 dpi with a range of 2 to 8 (Figures 2B,C). The divergence of T/F variants (compared to the inoculum) increased from 6 to 10 dpi (Figure 2A) due to *in vivo* virus replication. However, the composition of T/F virus lineages was not significantly changed (Figures 3, 4 and Figures S7, S8), which is consistent with previous report showing that the frequencies of major and minor T/F lineages remained relatively stable within 10 dpi (Kijak et al., 2017). In addition to the ORF intact sequences, 110 ORF defective sequences were observed in this study. We found that these defective sequences were phylogenetically co-localized with the functional variant in the inoculum (Data not shown), which is consistent with the previous finding (Yuan et al., 2017).

**Table 1:** Number of full length SIVmac251 *env* sequences obtained from different tissue compartments.

**Group**	**Macaque code**	**Tissue**	**Number of total sequences**	**Number of reading frame intact sequences**
6 dpi	070419	Rectum	32	29
group		Plasma	43	42
		Spleen	15	15
		Jejunum	36	33
		Descending colon	28	25
	070327	Rectum	61	44
		Plasma	33	30
		Spleen	24	22
		Jejunum	43	37
		Descending colon	34	32
	061127	Rectum	30	24
		Plasma	30	27
		Spleen	40	30
		Jejunum	38	34
		Descending colon	50	44
10 dpi	050429	Rectum	25	23
group		Plasma	28	25
		Spleen	24	23
		Jejunum	54	46
		Descending colon	43	42
	060319	Rectum	44	43
		Plasma	18	18
		Spleen	40	37
		Jejunum	47	46
		Descending colon	39	35
	060027	Rectum	33	28
		Plasma	44	42
		Spleen	48	46
		Jejunum	36	36
		Descending colon	35	27

*Totally 1,095 sequences were obtained by SGA, but only the reading-frame intact sequences (985) were used for downstream analysis. Only 7 out of 110 defective variants were found to be potentially caused by APOBEC-associated G-to-A hypermutation*.

### A cluster of rare T/F viruses was unequally represented in different tissue compartments

To further characterize the distribution of T/F viruses, we deconvoluted the phylogenetic relationships of T/F viruses derived from the different tissue compartments for each infected monkey. The analyses for 6 dpi monkeys are shown in Figure 3 and Figure S7 and analyses for 10 dpi monkeys are shown in Figure 4 and Figure S8. Overall, our data showed that in all monkeys the dominant T/F viruses (highlighted by right brackets) were consistently derived from the dominant variants of the inoculum (Figures 3, 4 and Figures S7, S8). Unexpectedly, we found that a cluster of rare T/F variants were transmitted at a much lower efficiency in each macaque (highlighted with red branches in Figures 3, 4 and Figures S7, S8). We found that the rare T/F virus cluster was phylogenetically related to 48 virus variants from the inoculum. Of these 48 identified from the inoculum, we were able to identify 44 of them shared by more than 4 out of 6 infected monkeys (Table S1). Therefore, we reasoned that this cluster of T/F viruses might derive from the same group of viruses in the inoculum. Interestingly, our data showed that this cluster of T/F viruses was unequally represented in different tissue compartments. Specifically, no viruses belonging to this cluster were identified in any infected rectum tissue. This indicates that either this cluster of SIV_mac251_ variants was absent from rectum or the frequency in rectum was much lower than in other tissue compartments.

### The unequally distributed T/F variants exhibited animal-specificity and high CG dinucleotide frequency

**Figure 5:**
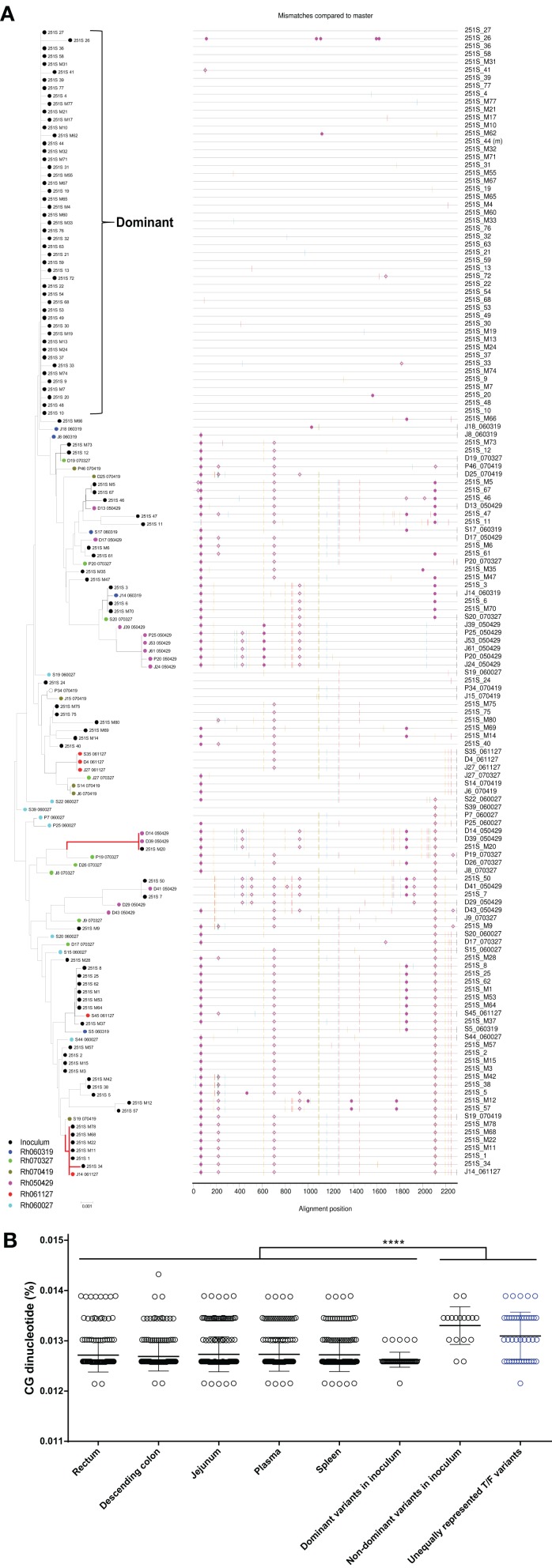
The unequally represented T/F variants were animal-specific and their average CG dinucleotide frequency was high. **(A)** The unequally represented T/F variants identified in Figures 3, 4, and Figures S7, S8 were pooled together with inoculum virus sequences for N-J phylogeny and Highlighter analyses. Identical variants identified at both the descending colon/jejunum and the inoculum are highlighted with red branches. **(B)** Mean CG dinucleotide frequencies of the T/F and inoculum viruses were also calculated and compared. The data showed this cluster of unequally represented T/F variants had significantly higher average CG frequency.

To clarify whether the unequally represented T/F variants (highlighted with red branches in Figures 3, 4 and Figures S7, S8) were shared among infected macaques, we pooled these *env* sequences together with inoculum virus sequences and performed phylogenetic analysis. The results confirmed that all T/F variants were phylogenetically related to the non-dominant variants in inoculum. However, no shared variant was found among different monkeys (Figure 5A), indicating that the transmission of these variants might take place in a stochastic way. Furthermore, the frequencies of these variants were low in all monkeys. This implied that their replication capacities might be lower than the dominant variants and therefore their transmission efficiencies were restricted, especially in rectum mucosa. To unveil the difference between the dominant and unequally represented T/F variants, we performed CG dinucleotide frequency analysis. Our data showed that the average CG dinucleotide frequency of these unequally represented T/F variants was significantly higher (*P* < 0.0001) than that of T/F viruses in different tissues and the dominant variants in inoculum, but was slightly lower than the average CG frequency of the non-dominant variants in inoculum (Figure 5B). High CG dinucleotide frequency within the *env* gene was recently demonstrated to be deleterious to HIV replication (Takata et al., 2017). Our data further suggested that host CG suppression might also play a role in transmission bottleneck selection. Additionally, identical SIVmac251 variants belonging to this cluster were detected simultaneously in descending colon/jejunum and the inoculum in 2 out of 6 macaques (highlighted with red branches in Figure 5A).

## Discussion

Epidemiological observation suggested that the risk of HIV transmission was higher for anorectal exposure than vaginal exposure(Patel et al., 2014), which might be due to the differences of transmission bottleneck and infection multiplicity (Li et al., 2010; Tully et al., 2016) between modes of sexual transmission. As the transmission of multiple founder variants might associate with faster HIV disease progression (Janes et al., 2015), we hypothesized that characterizing the T/F viruses under a high multiplicity infection setting could provide new insights into the mechanisms of HIV/SIV anorectal transmission. In current study, we employed a single high dose SIV_mac251_ challenge model in Chinese rhesus macaques to characterize T/F viruses. This model has previously been shown to be able to establish infection by multiple founder viruses in Indian rhesus macaques (Liu et al., 2010). Specifically, we challenged 6 rhesus macaques intrarectally with 3.1 × 10^4^ TCID_50_/monkey of SIV_mac251_ and euthanized the animals for virus detection at 6 or 10 dpi. A recent study showed dramatic shifts in the frequencies of the viruses that composed the HIV-1 population within hosts infected by multiple viral lineages (Kijak et al., 2017), suggesting that detection at the very early phase of infection may provide a more accurate view of the T/F viruses. We found that the number of T/F viruses in peripheral blood ranged from 2 to 8, which was comparable with previously published data (Liu et al., 2010) and closely resembled the clinical data showing that 2–10 founder viruses could identified in multivariant transmissions (Li et al., 2010). The dominant T/F variants came from the dominant variants in the inoculum, and were identified in all tissues of all infected monkeys. The high transmission efficiency may be due to the significantly lower CG dinucleotide frequencies in the *env* gene (Figure 5B), as a previous finding suggested that high CG content in the *env* gene was deleterious to HIV replication (Takata et al., 2017). Our findings are inconsistent with a previous study which suggested that founder viruses were animal-specific and primarily derived from rare variants in the inoculum (Yuan et al., 2017). This discrepancy is possibly caused by differences between macaques (Chinese vs. Indian) and SIV_mac251_ inoculums used in the two studies. In addition to peripheral blood, we also analyzed the *env* sequences obtained from rectum, descending colon, jejunum, and spleen. To our surprise, the data showed that rectum (the inoculation site) was not the tissue that comprised the most diverse T/F variants. Although no statistical significance was reached, the number of T/F viruses observed in rectum tended to be lower than other tissue compartments. This finding was corroborated by the observation that a cluster of rare T/F viruses was not detected in rectum tissues of any monkeys, but was identified in the descending colon, jejunum, spleen, or plasma. Compartmentalization of HIV-1 in chronically infected patients and *in vivo* cell-to-cell transmission have been extensively reported (van Marle et al., 2007; Bull et al., 2009; Schnell et al., 2009; Bednar et al., 2015; Law et al., 2016). However, our study is the first to indicate that the distribution of T/F viruses is different among tissues in very early SIV rectal transmission.

To further delineate the T/F viruses in different tissue compartments, we analyzed the phylogenetic relationships of SIV_mac251_ *env* sequences for each monkey and found that a cluster of rare T/F viruses was transmitted at low efficiency in all monkeys (highlighted with red branches in Figures 3, 4 and Figures S7, S8), which was unequally represented in different tissue compartments. Most strikingly, it was absent from rectum tissues in all 6 macaques. This result indicates that rectum is less susceptible to the infection of this cluster of variants. It also implies that rectum may not be the only site of SIV entry under this particular experiment setting, which is consistent with previous studies showing that multi-site entry was possible after intra rectal exposure (Ribeiro et al., 2011; Smedley et al., 2014).

SIV can disseminate very rapidly after mucosal inoculation (Ribeiro et al., 2011; Barouch et al., 2016), which may explain our finding that the dominant variant cluster presented in all tissues of all monkeys (Figures 3, 4 and Figures S7, S8). However, the finding that some rare viruses were not detectable in rectum could not be decidedly explained by rapid *in vivo* dissemination, because T/F viruses usually need to be expanded locally before dissemination (Li et al., 2005; Stone et al., 2010) and it is very unlikely that locally expanded viruses migrate to distal sites with no evidence of the expansion at the portal of entry. Under our experiment setting, possibility of virus entry via descending colon mucosa could not be completely excluded as we found that a rare SIVmac251 variant was shared only by descending colon and inoculum in Rh050429.

Although a relatively large number of *env* sequences were generated in our study (985 reading-frame intact sequences in total; on average 31, 38, and 34 sequences were obtained from rectum, descending colon, and jejunum for each monkey, (Table 1), the sampling depth could not ensure that we have captured the entire pool of T/F variants. Therefore, some very rare variants may have potentially been missed. In spite of this limitation, our observations did demonstrate that a rare T/F lineage was unequally represented between rectum and other tissues at the very early phase of rectal SIV infection and suggested that CG dinucleotide host suppression might contribute to the bottleneck selection.

## Author contributions

YW, JX, and QL conceptualized and designed the study. JC, YW, YR, LL, QW, and GK performed all experiments. YW, JC and LD analyzed the data. YW, JC, and LD wrote the manuscript with intellectual input from all authors.

### Conflict of interest statement

The authors declare that the research was conducted in the absence of any commercial or financial relationships that could be construed as a potential conflict of interest.
