# Supplementary figures and images for: Identification of Unequally Represented Founder Viruses Among Tissues in Very Early SIV Rectal Transmission
Source: Front Microbiol. 2018 Mar 29;9:557. doi: 10.3389/fmicb.2018.00557 (PMC5884942; doi:10.3389/fmicb.2018.00557)

Fig.S1A Rh061127\_Rectum

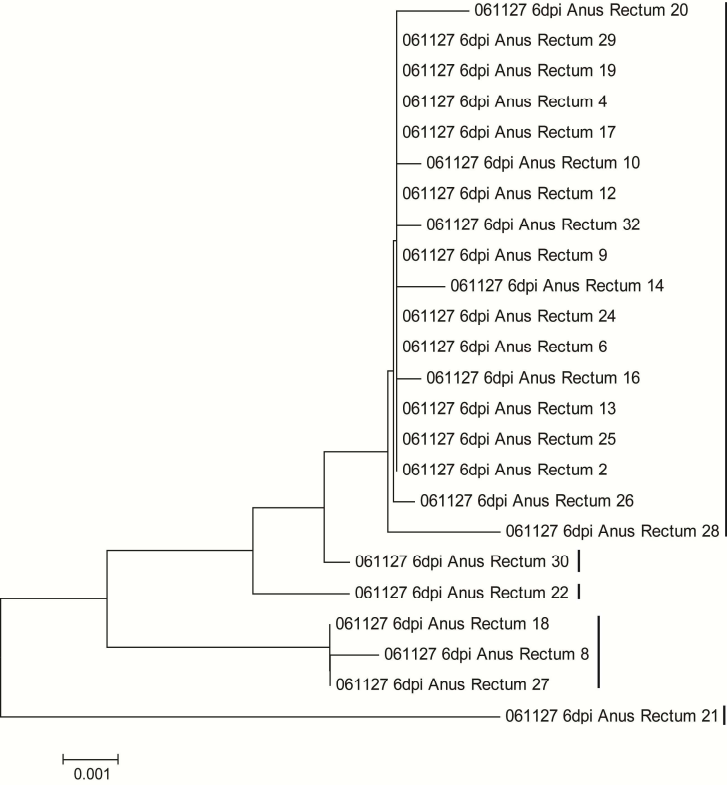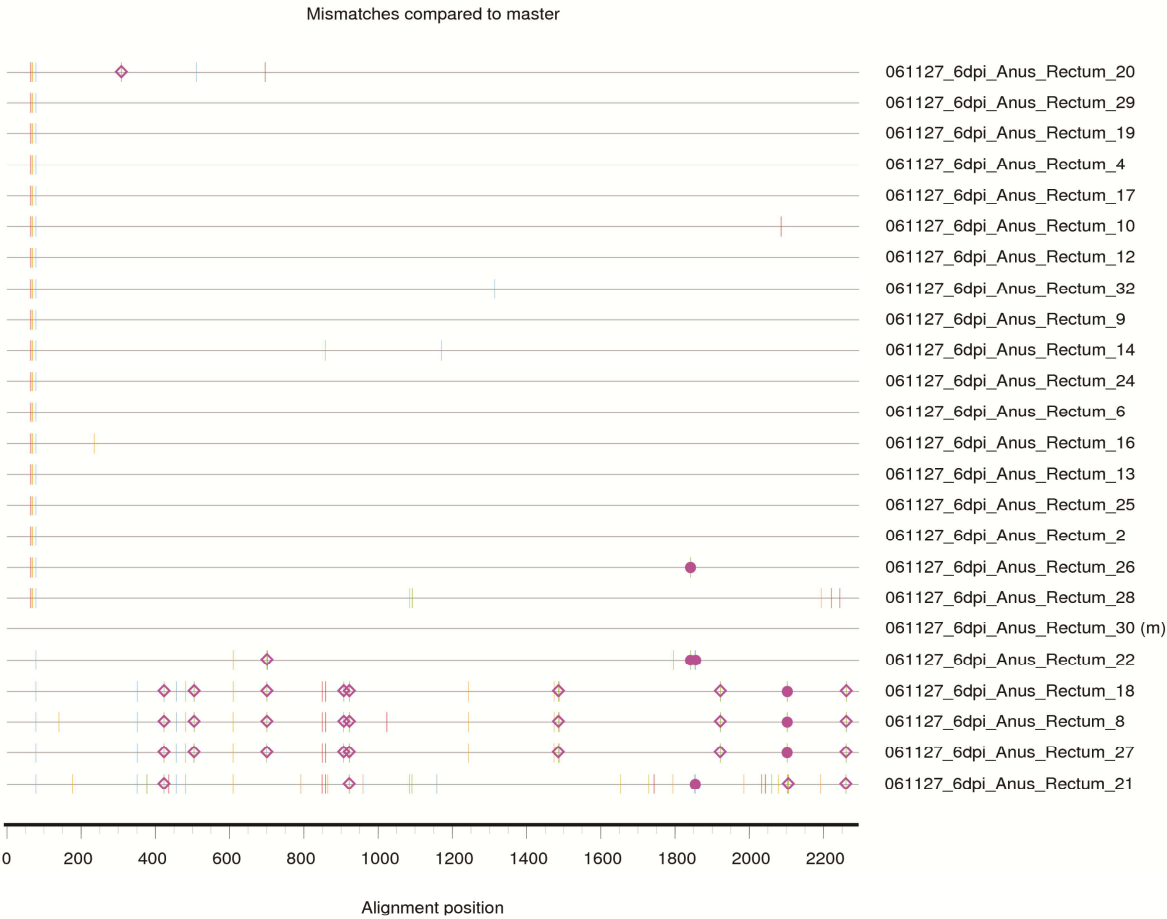

Fig.S1B Rh061127\_Descending colon

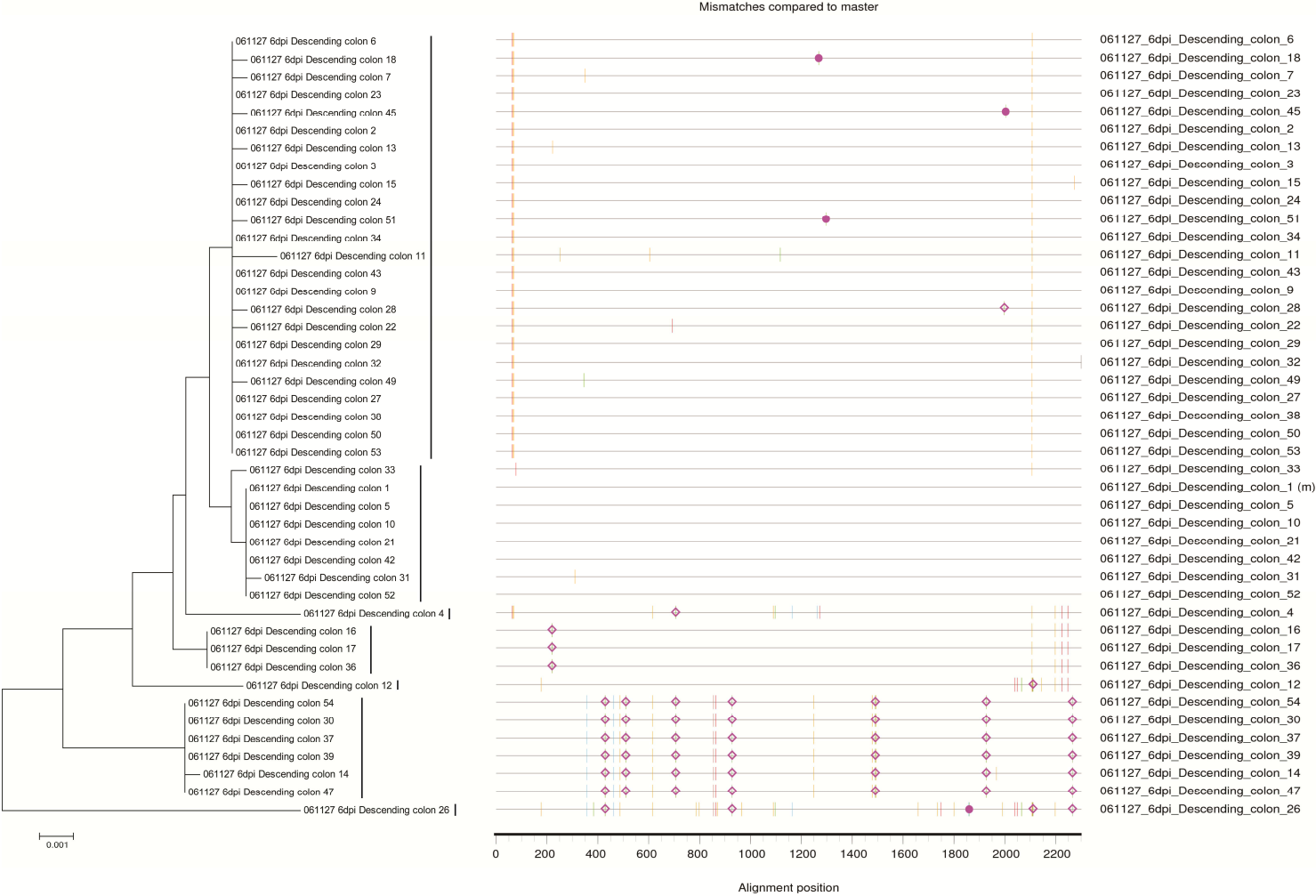

Fig.S1C Rh061127 Jejunum

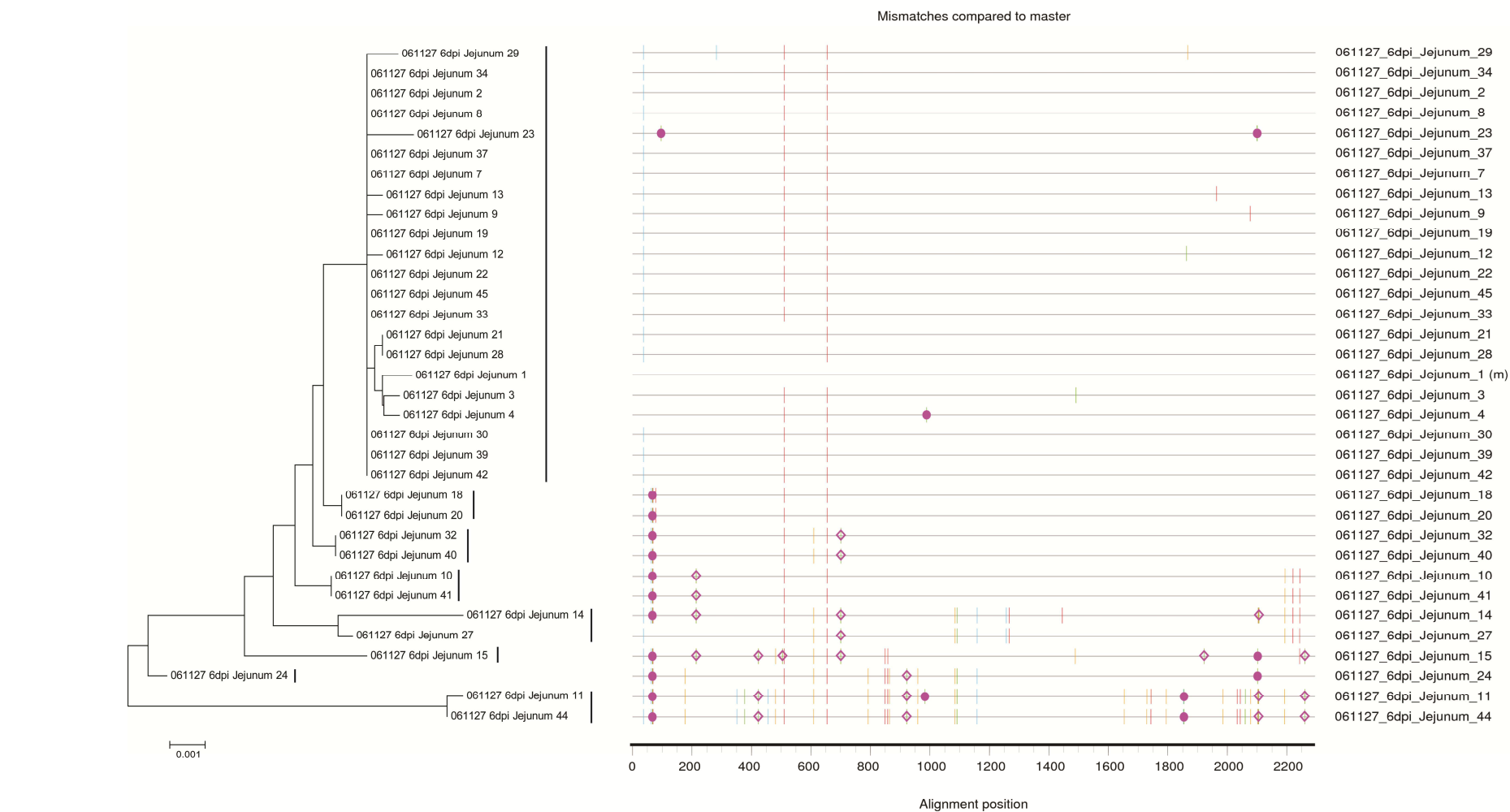

Fig.S1D Rh061127\_Plasma

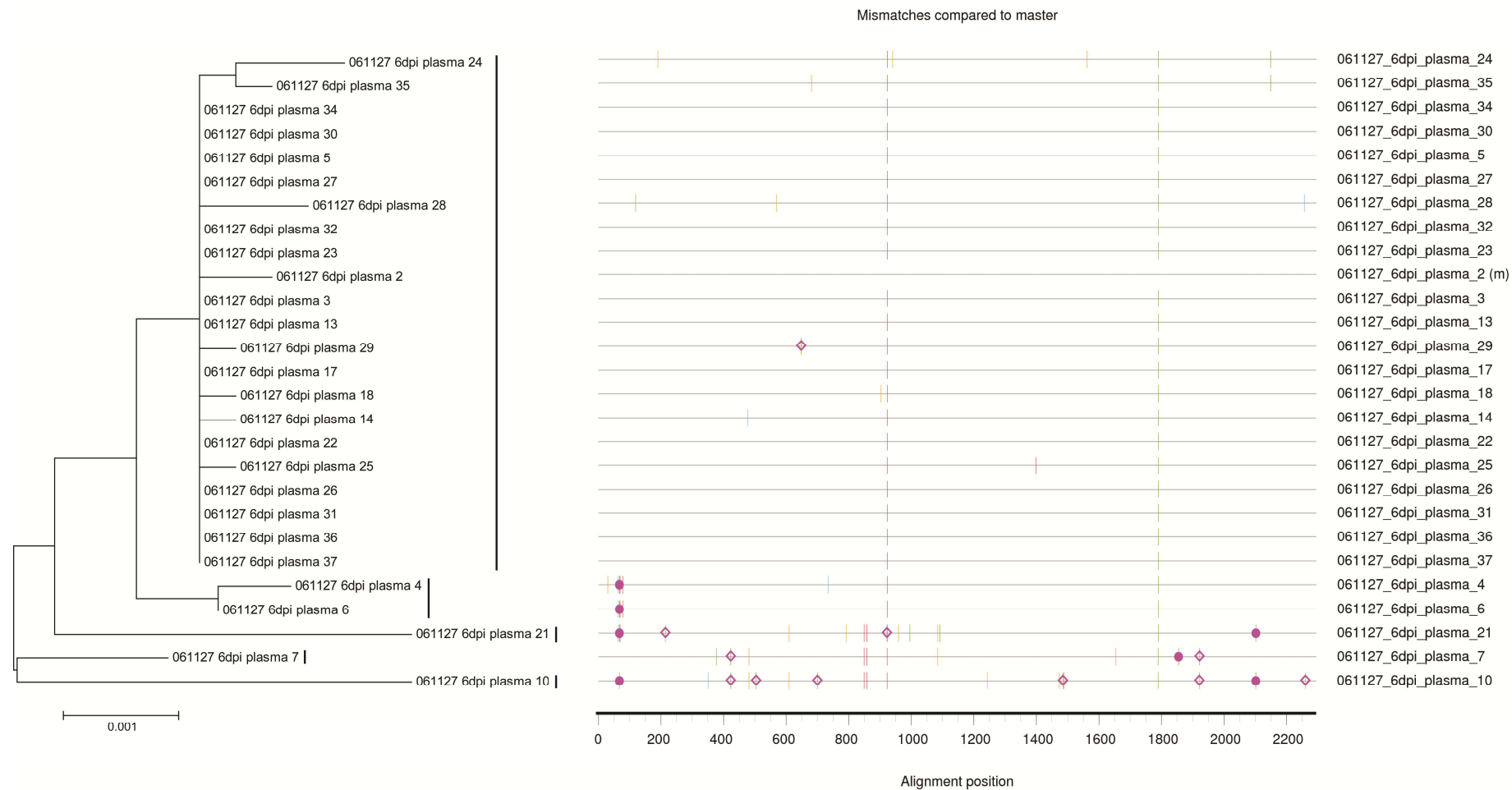

Fig.S1E Rh061127\_Spleen

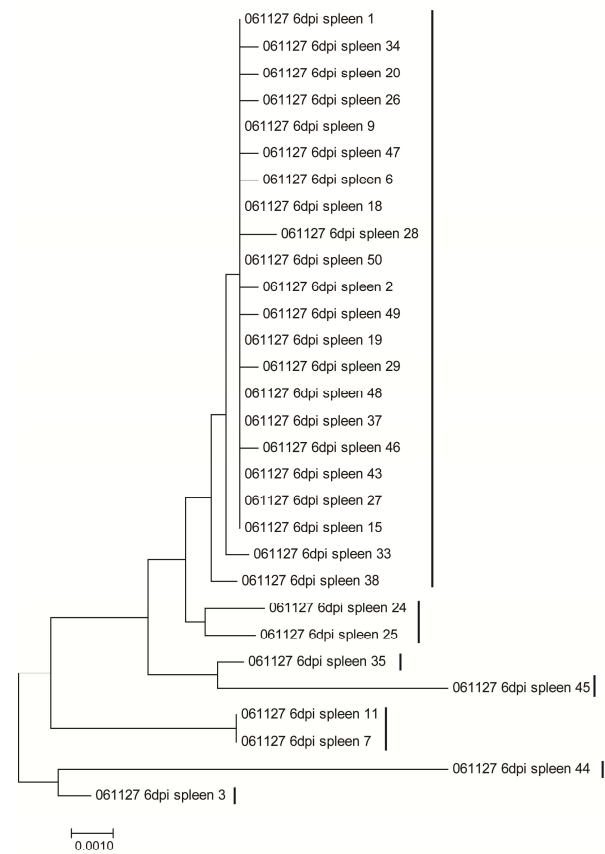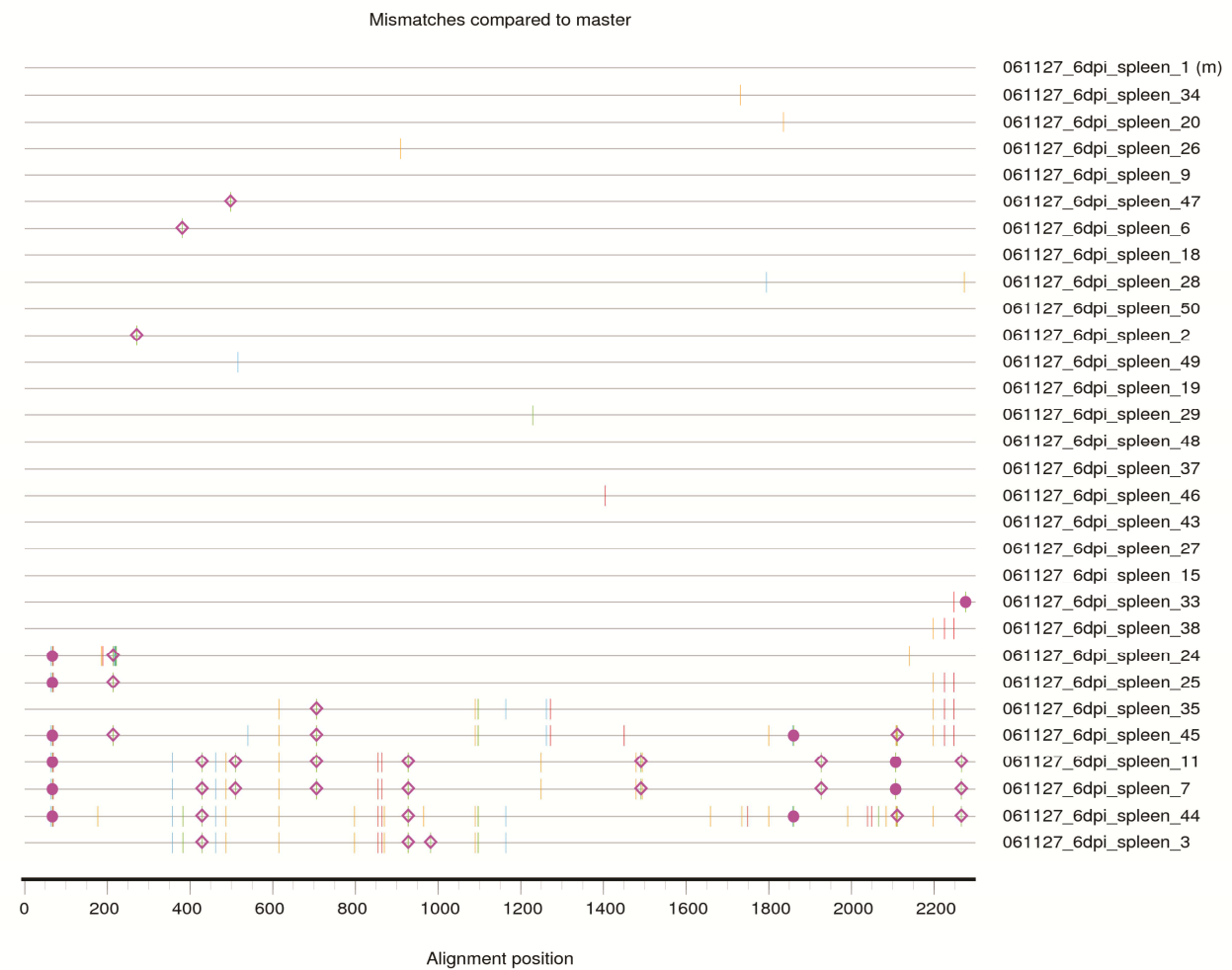

Supplement: Supplementary file 1 [file Image1.PDF]

Fig.S2A Rh070327\_Rectum

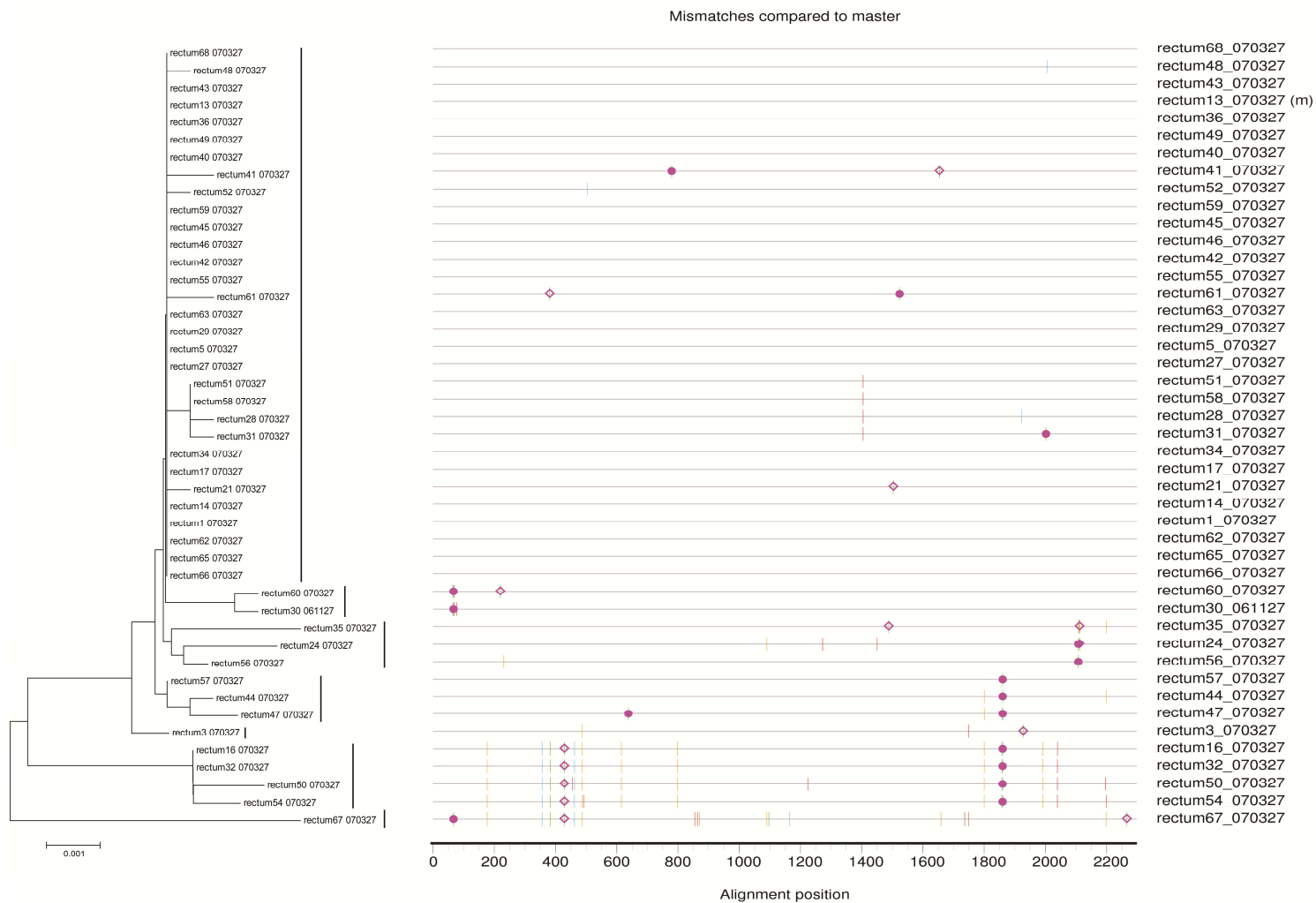

Fig.S2B Rh070327\_Descending colon

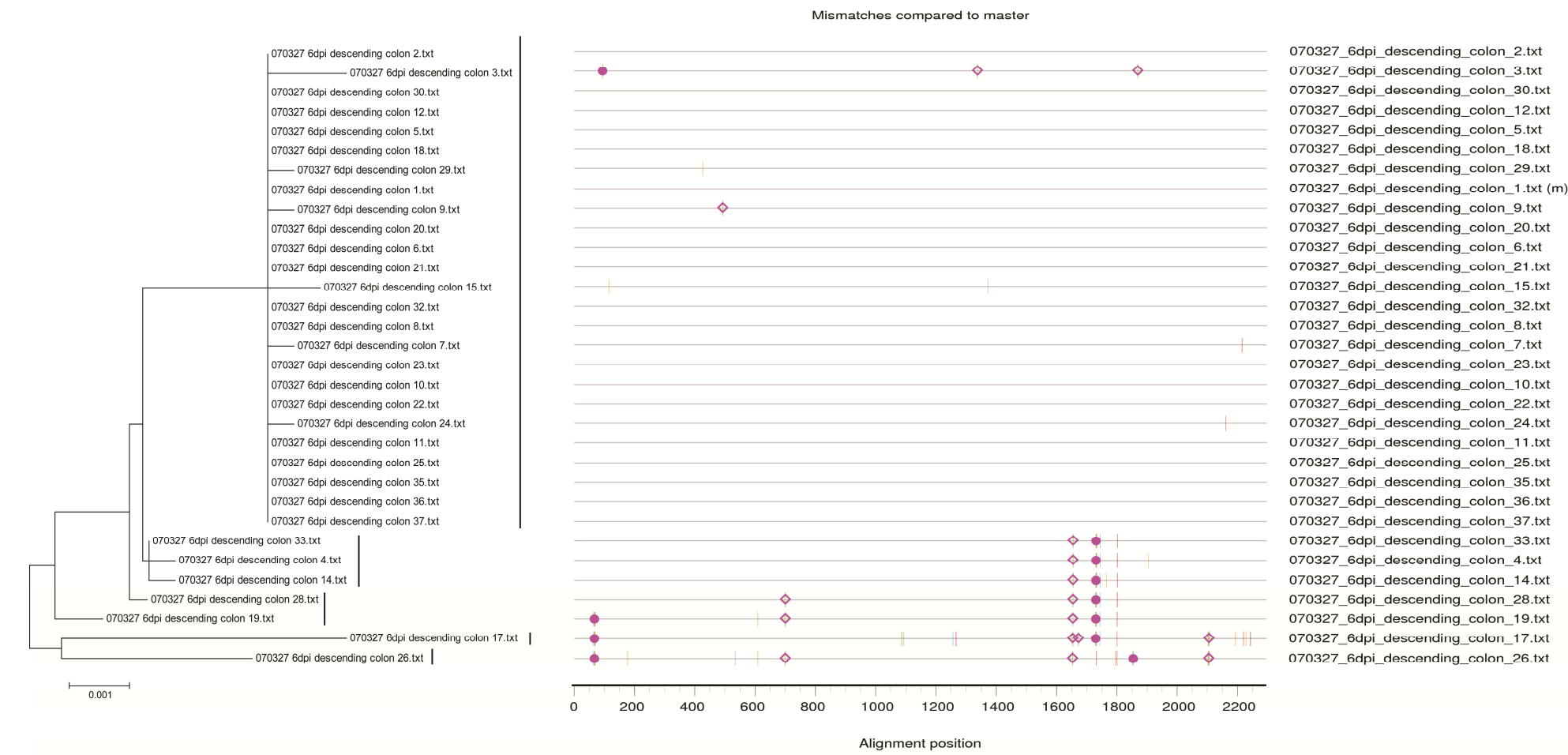

Fig.S2C Rh070327\_Jejunum

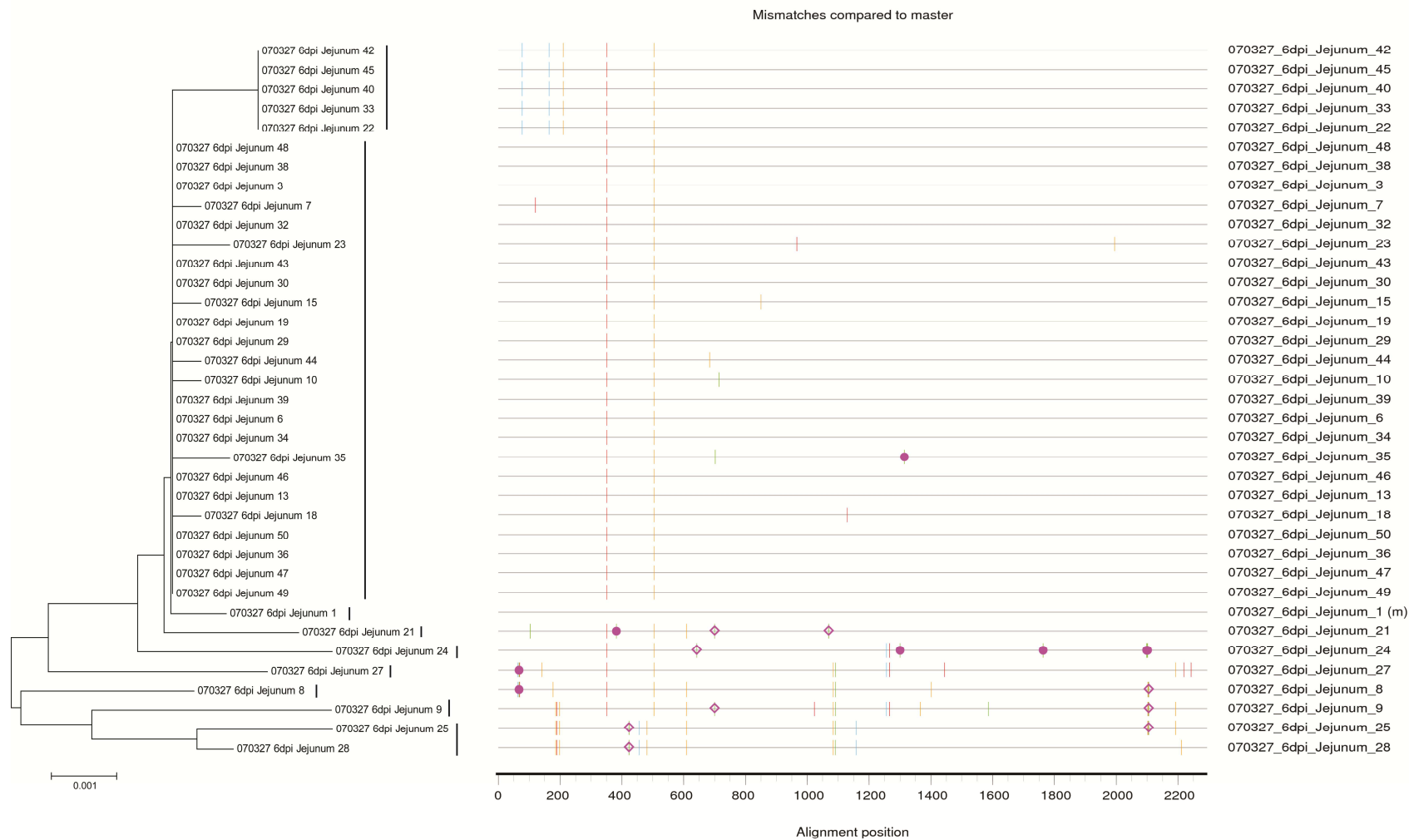

Fig.S2D Rh070327\_Plasma

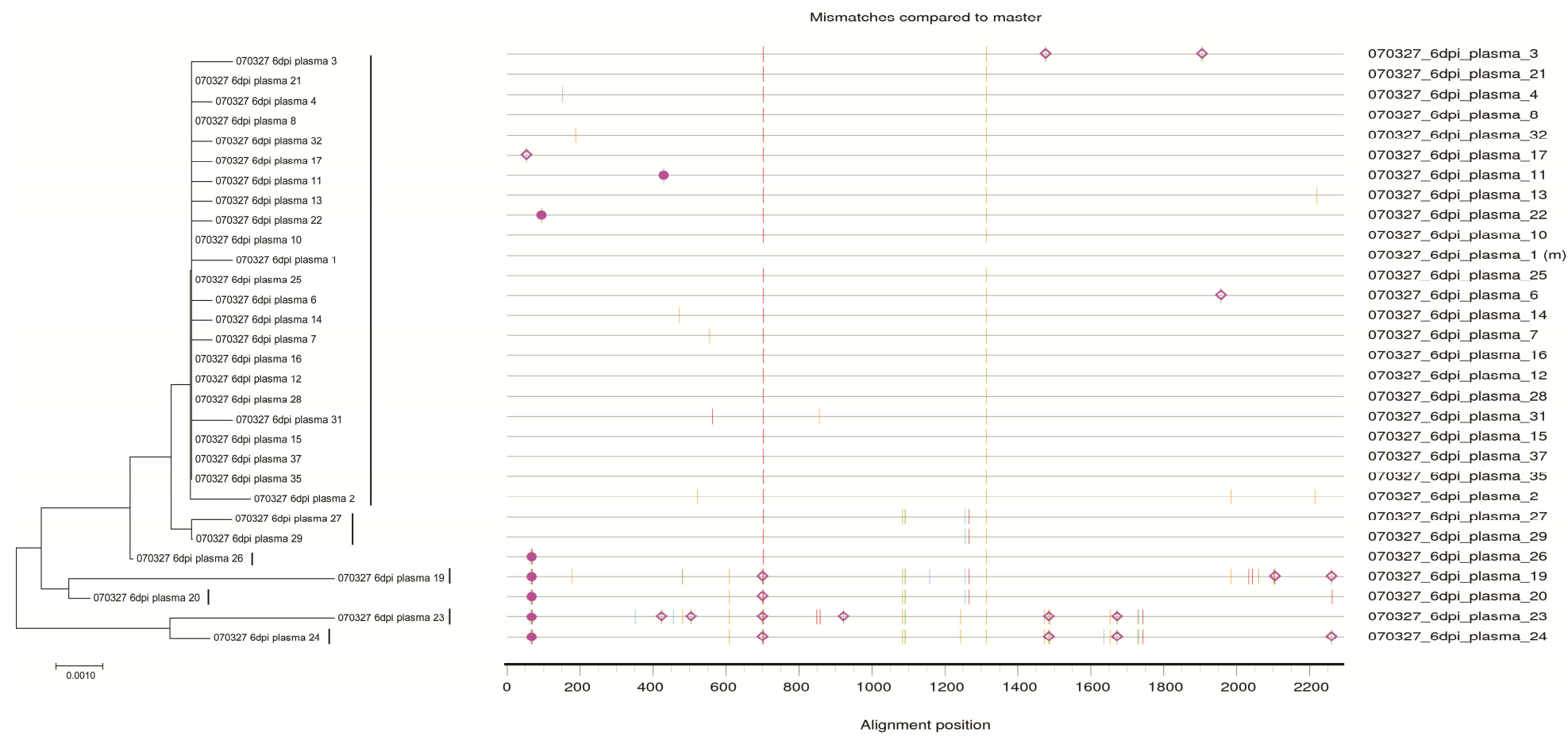

Fig.S2E Rh070327\_Spleen

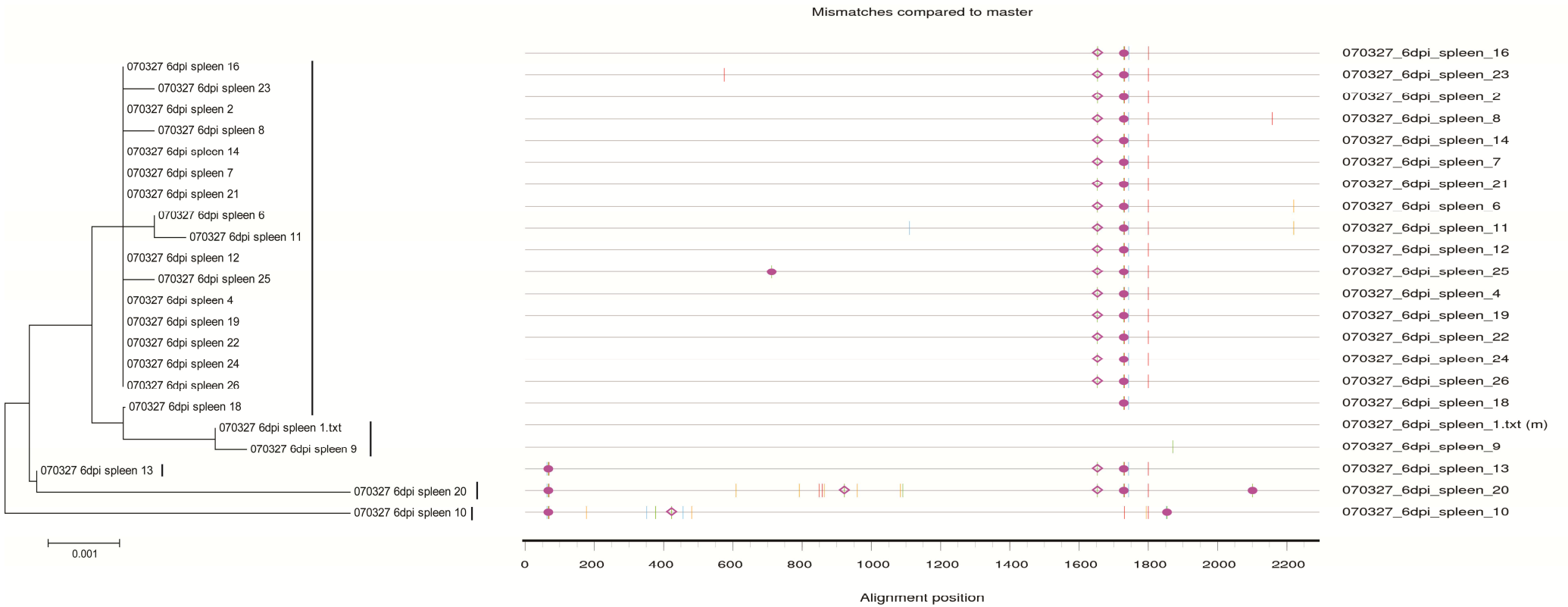

Supplement: Supplementary file 2 [file Image2.PDF]

Fig.S3A Rh070419\_Rectum

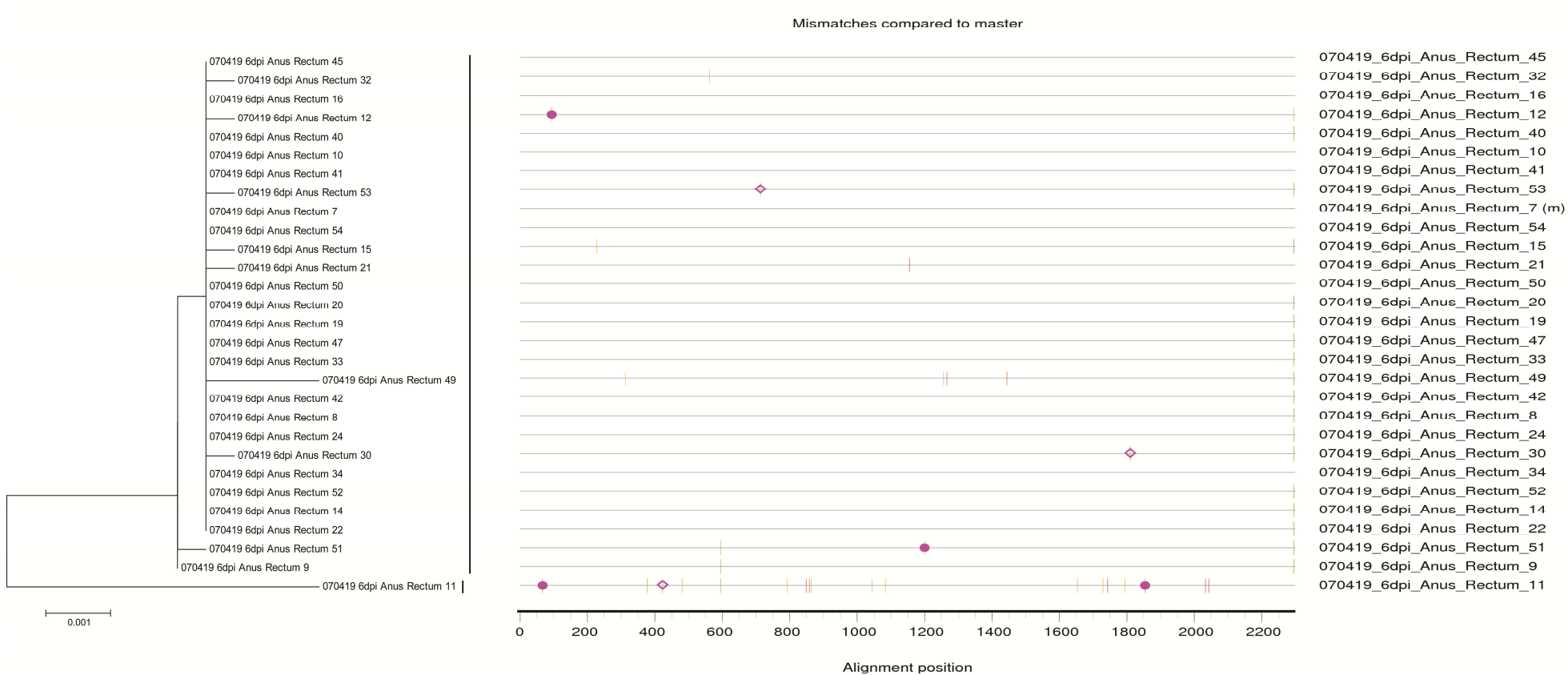

Fig.S3B Rh070419\_Descending colon

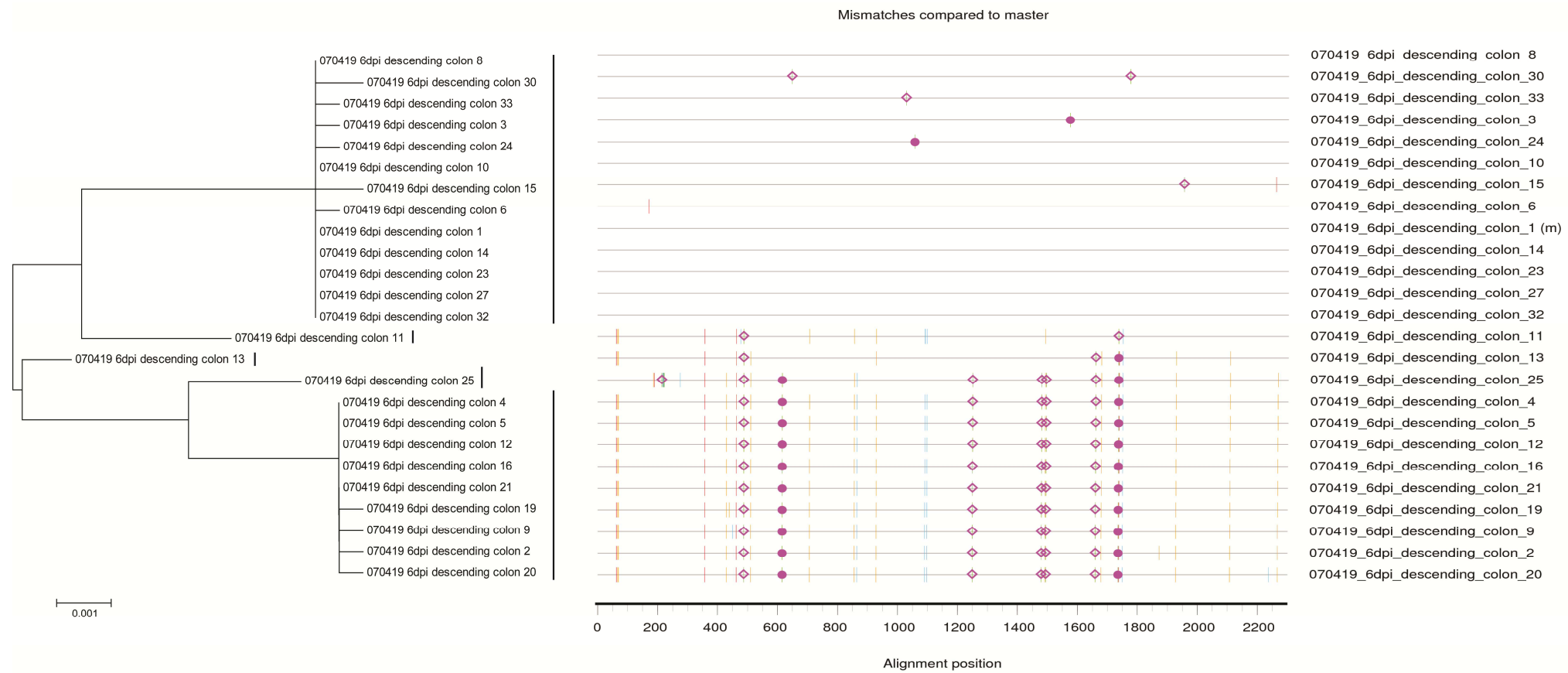

Fig.S3C Rh070419\_Jejunum

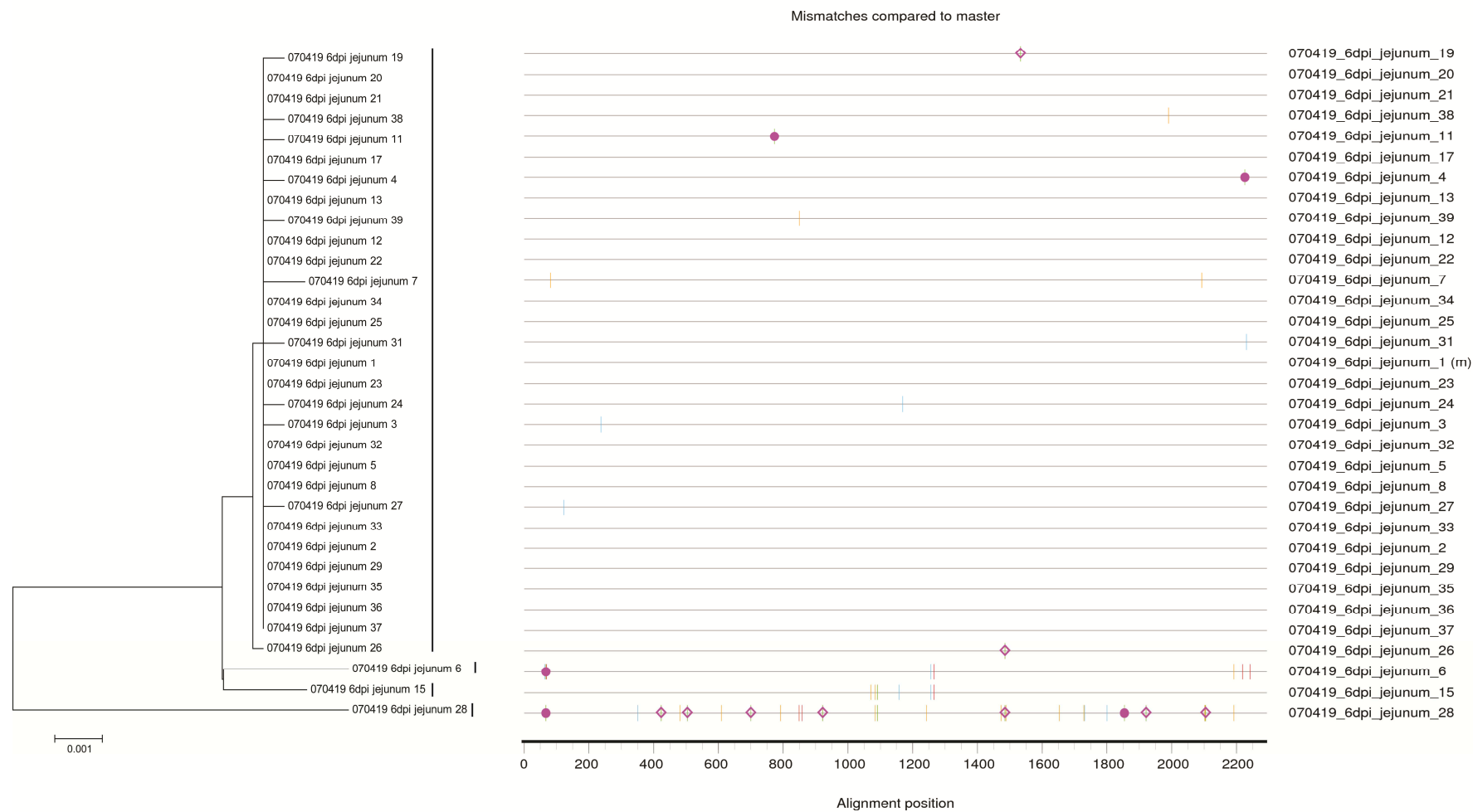

Fig.S3D Rh070419 Plasma

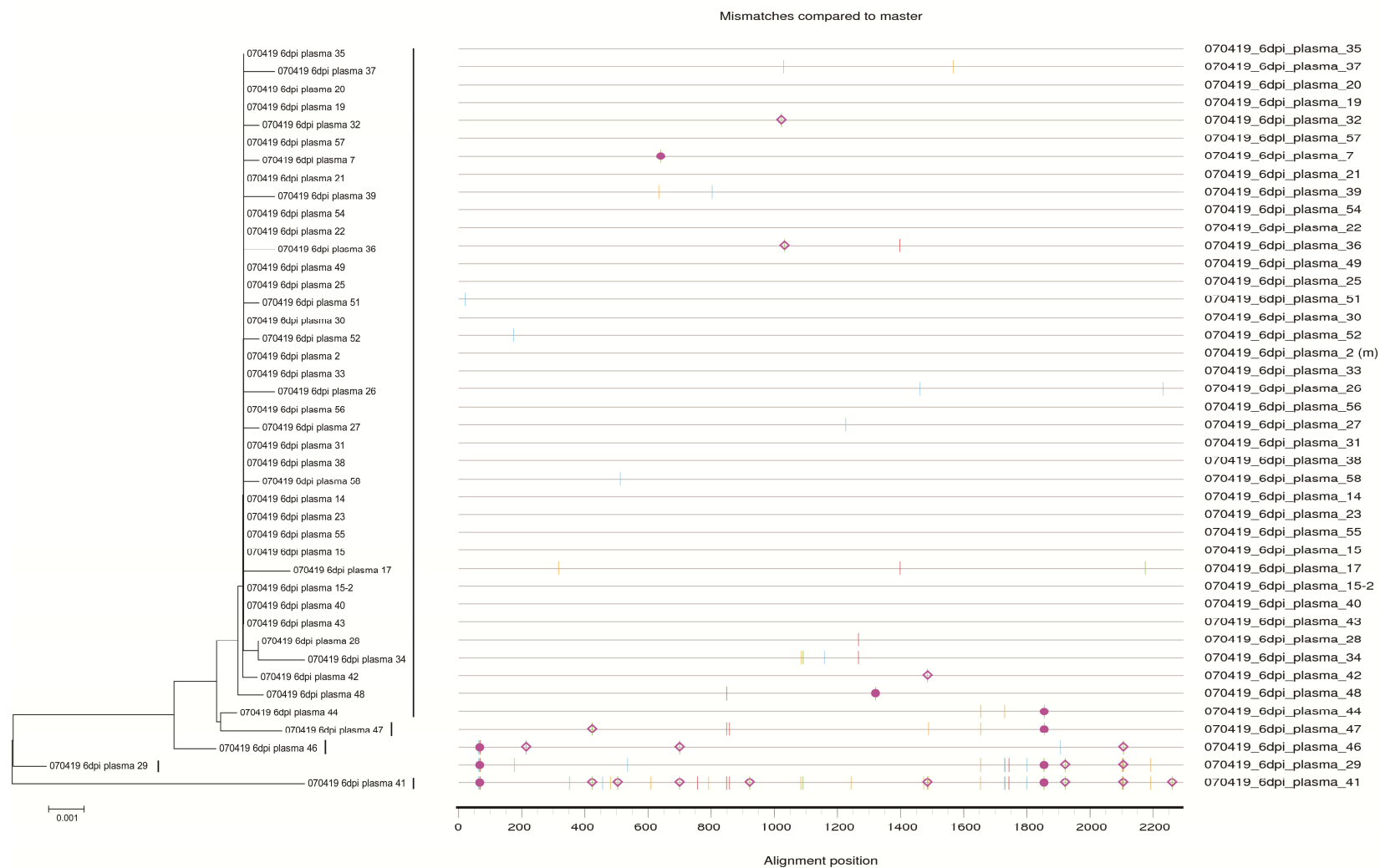

Fig.S3E Rh070419\_Spleen

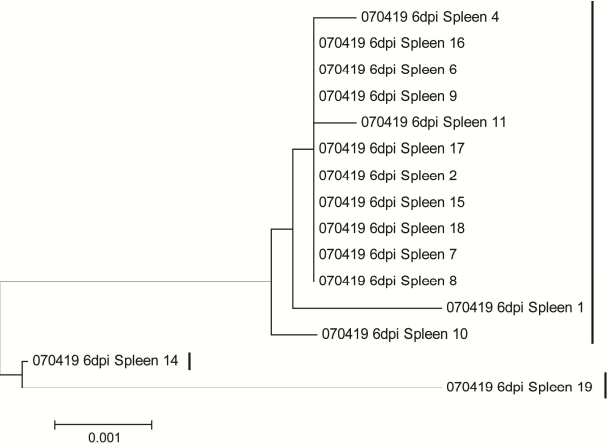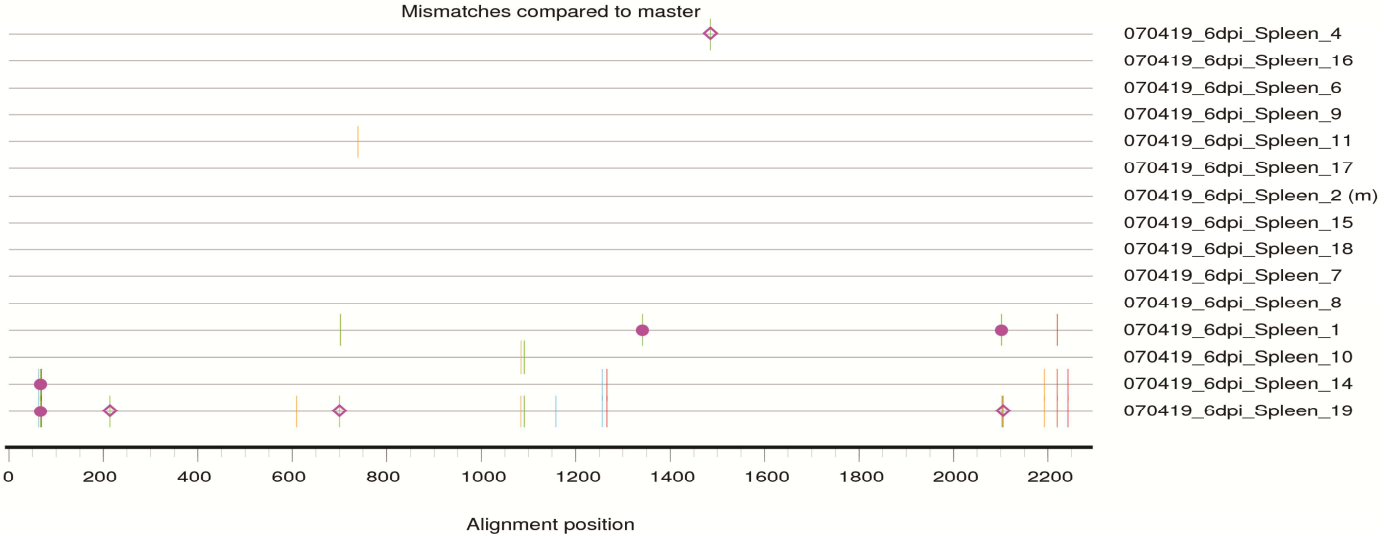

Supplement: Supplementary file 3 [file Image3.PDF]

Fig.S4A Rh050429\_Rectum

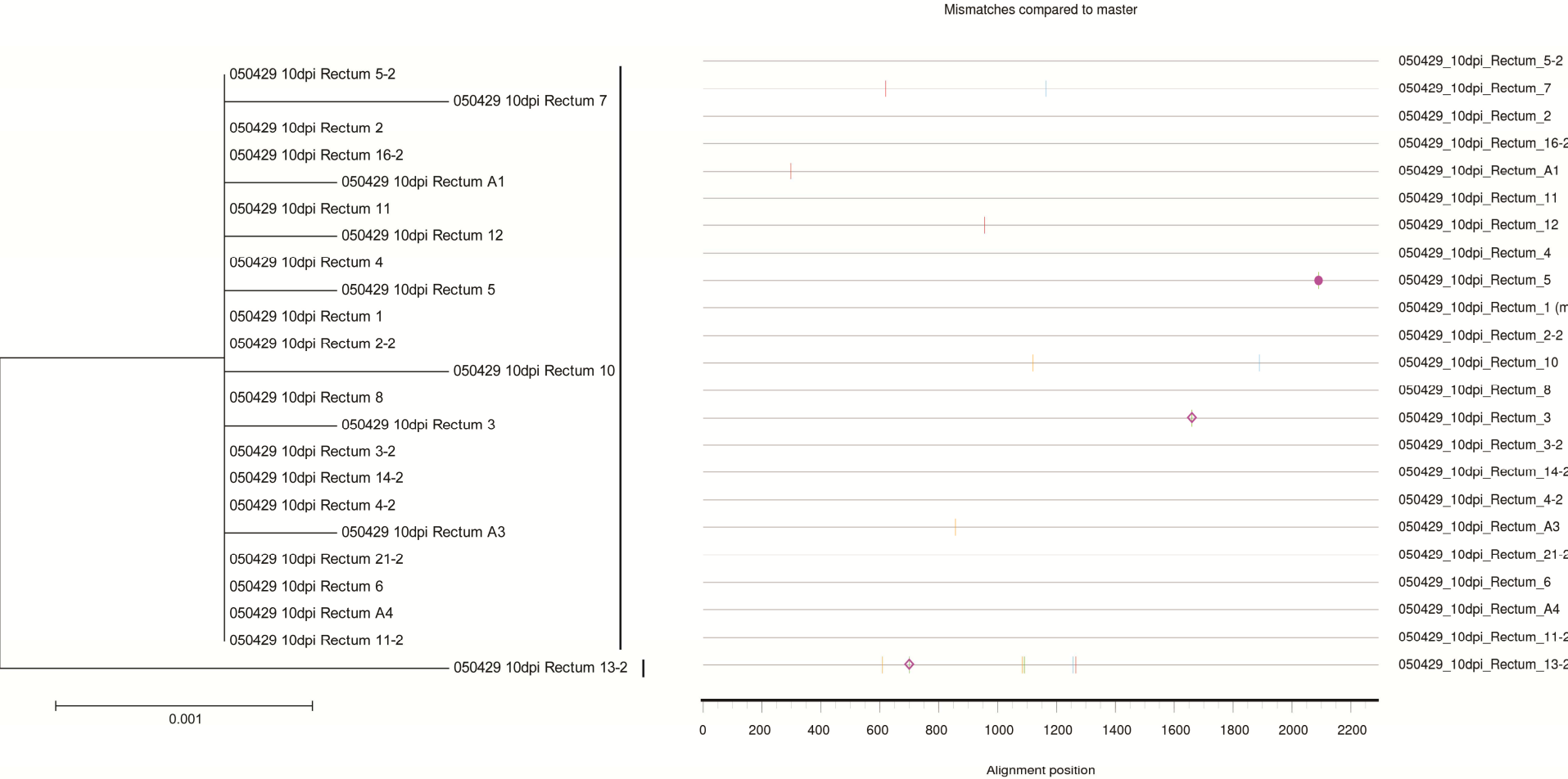

Fig.S4B Rh050429\_Descending colon

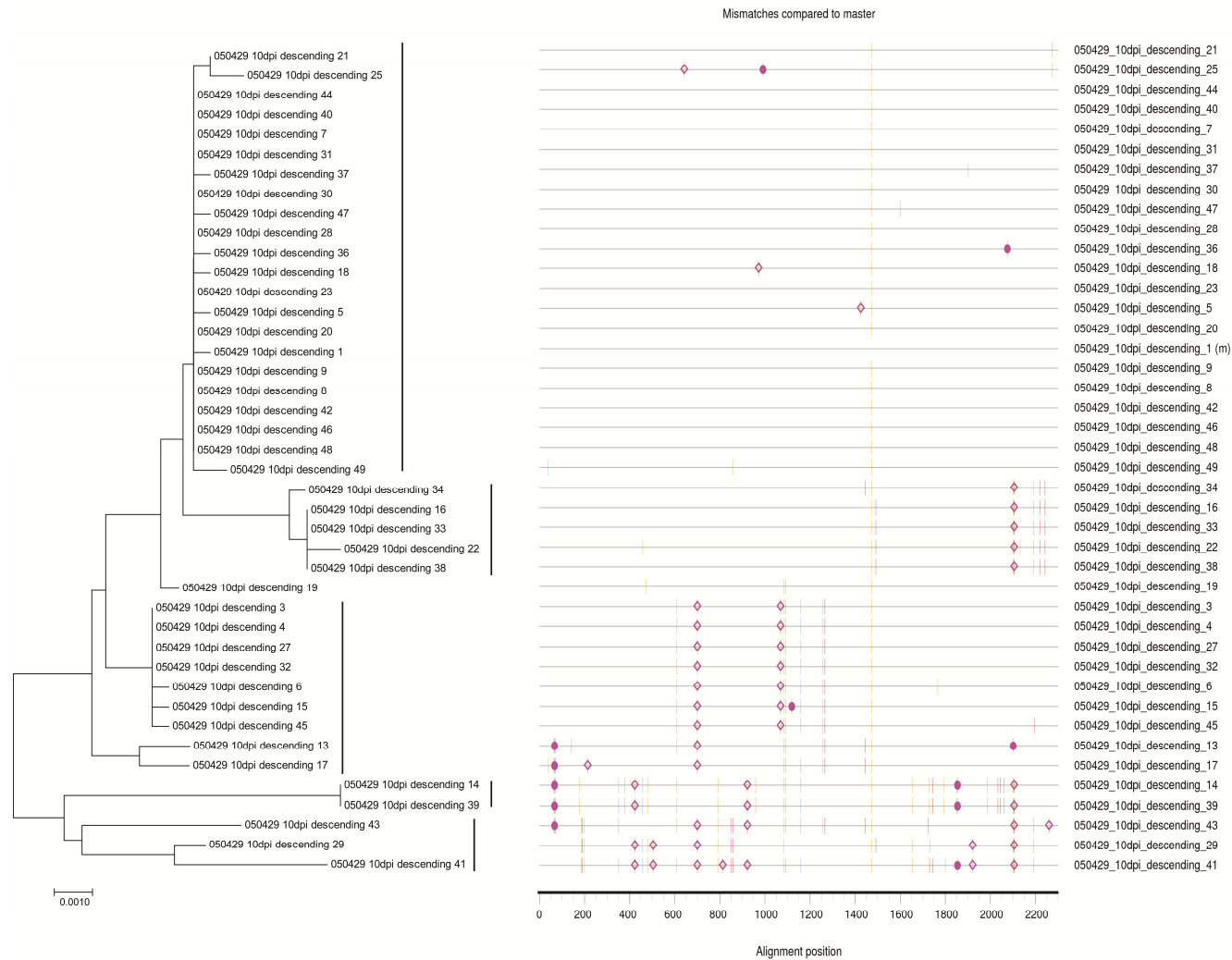

Fig.S4C Rh050429\_Jejunum

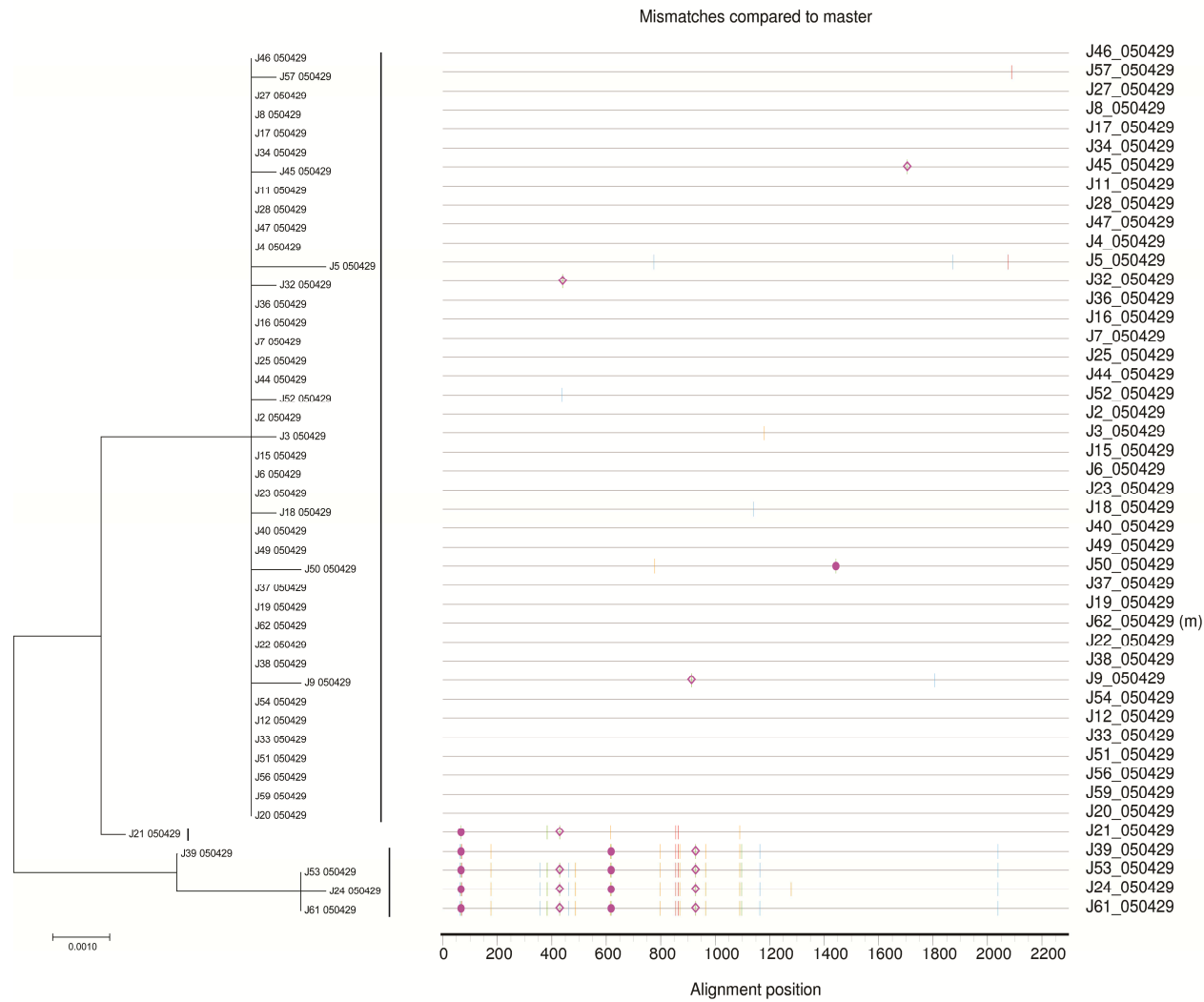

Fig.S4D Rh050429\_Plasma

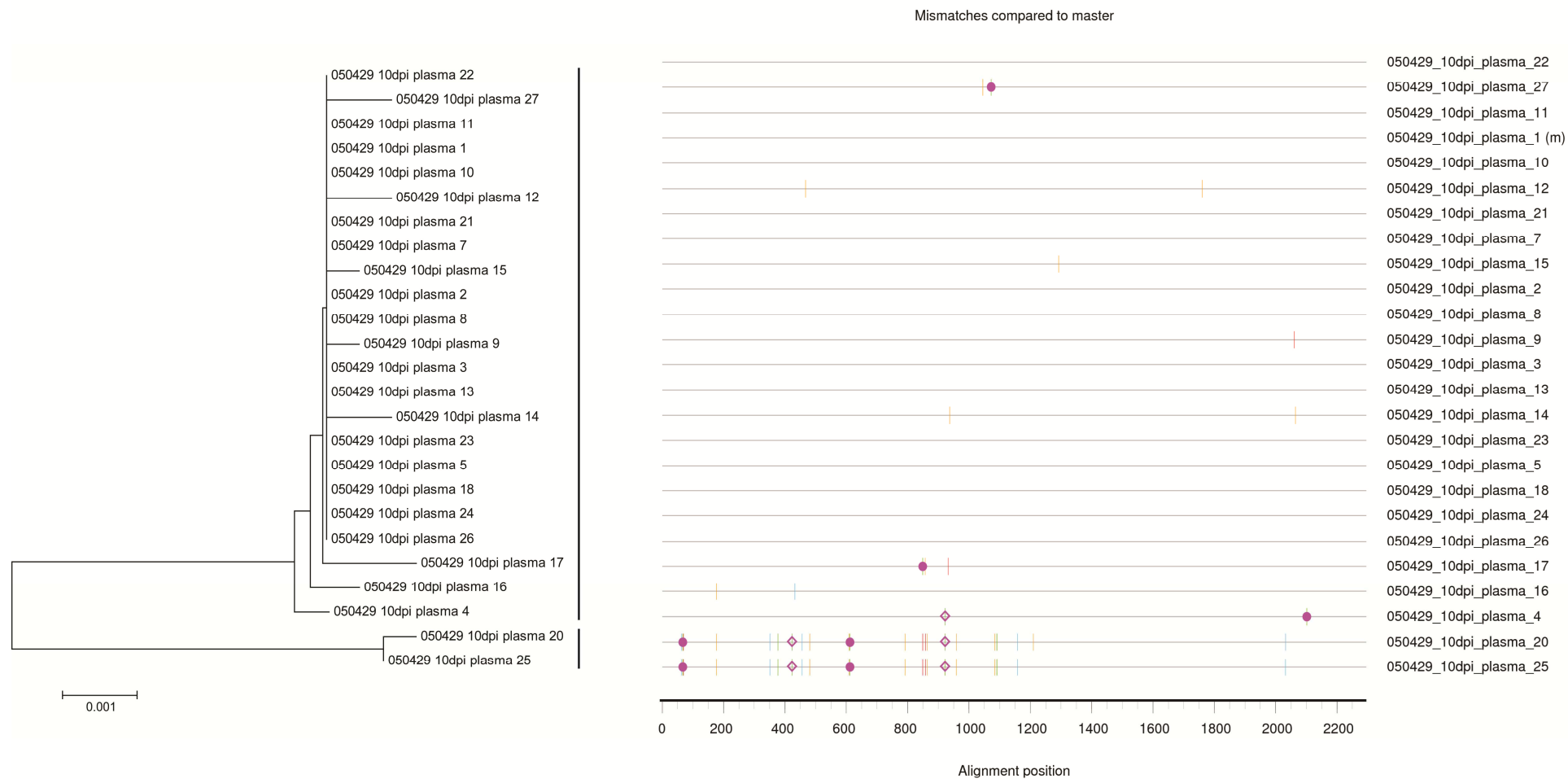

Fig.S4E Rh050429\_Spleen

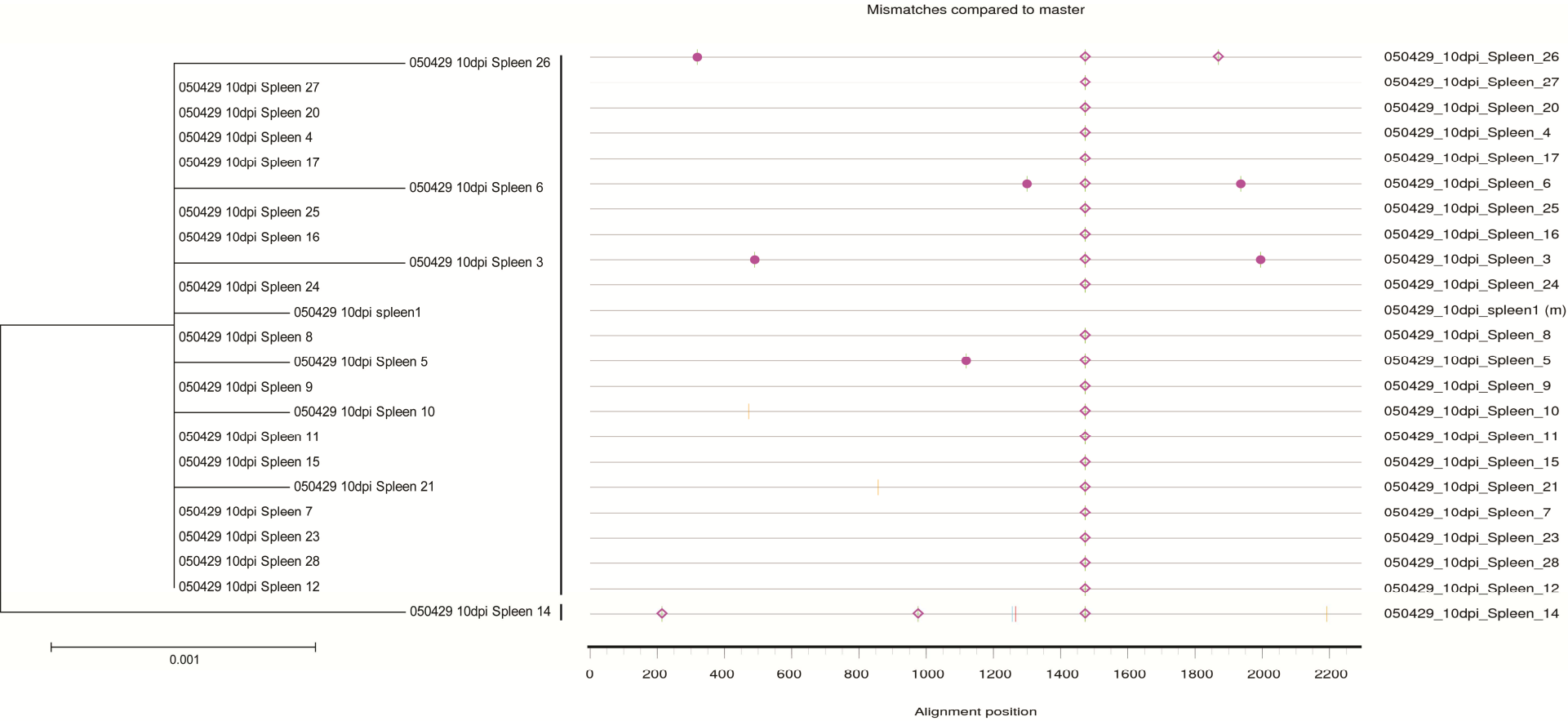

Supplement: Supplementary file 4 [file Image4.PDF]

Fig.S5A Rh060027\_Rectum

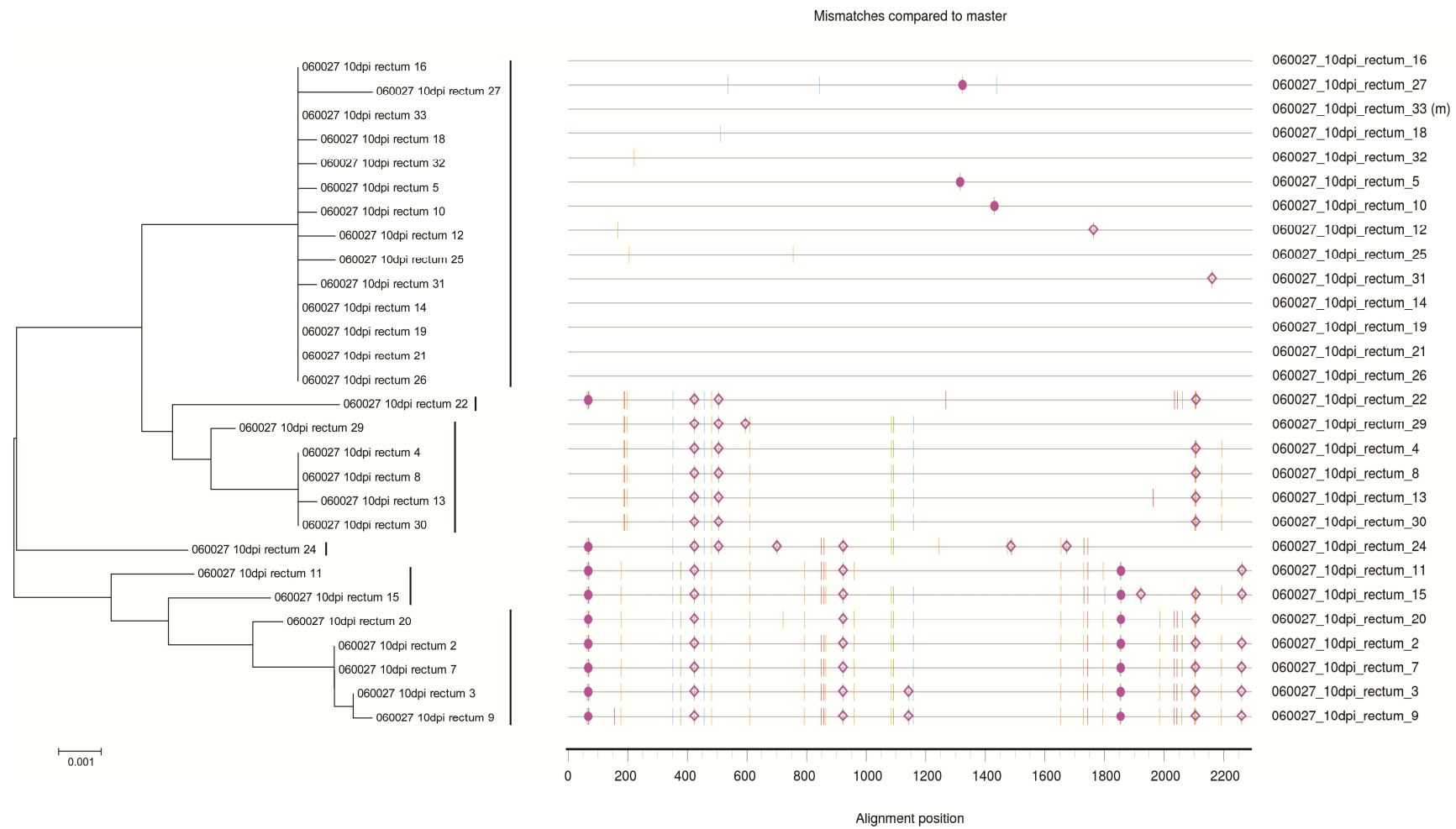

Fig.S5B Rh060027\_Descending colon

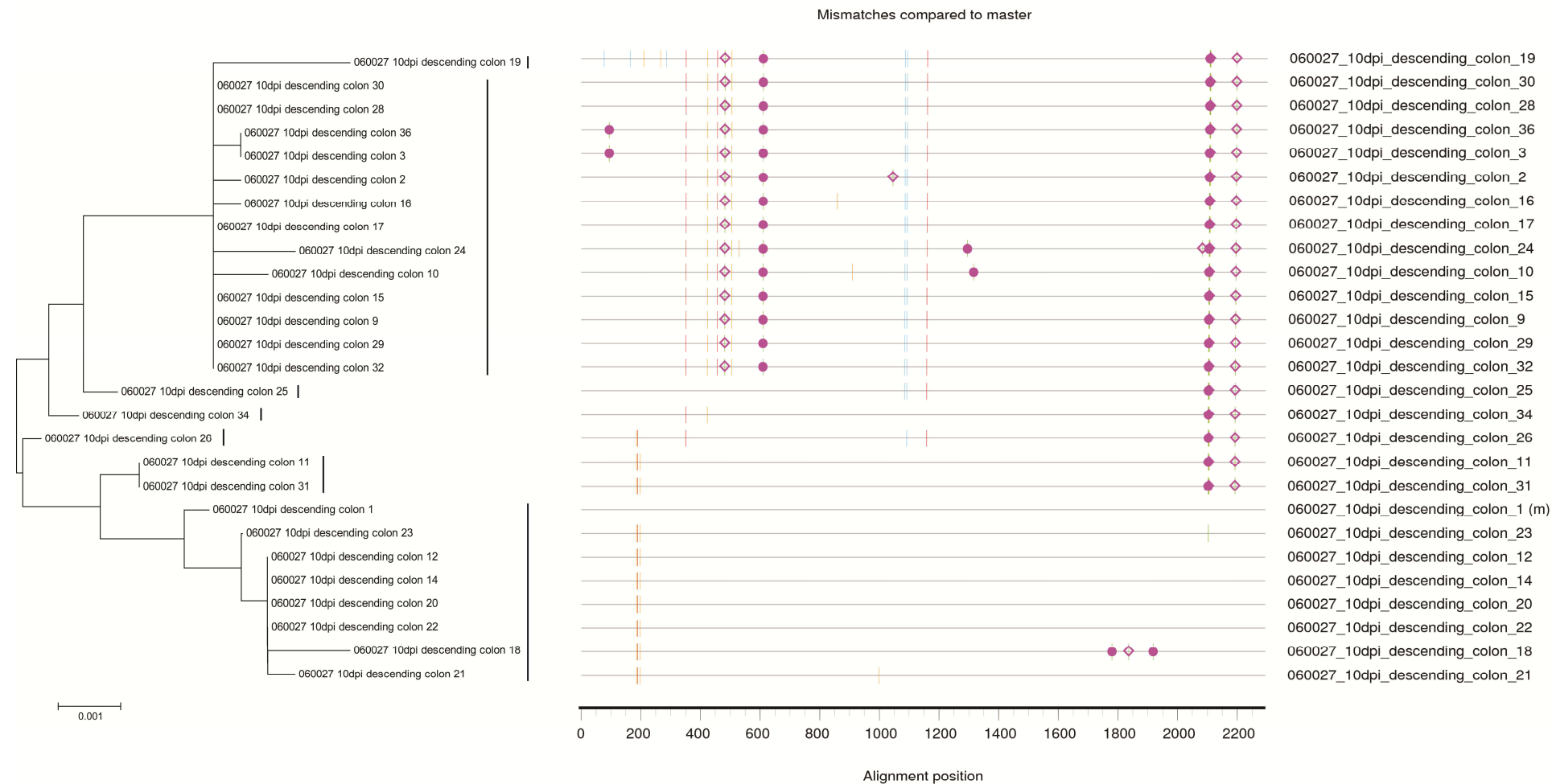

Fig.S5C Rh060027\_Jejunum

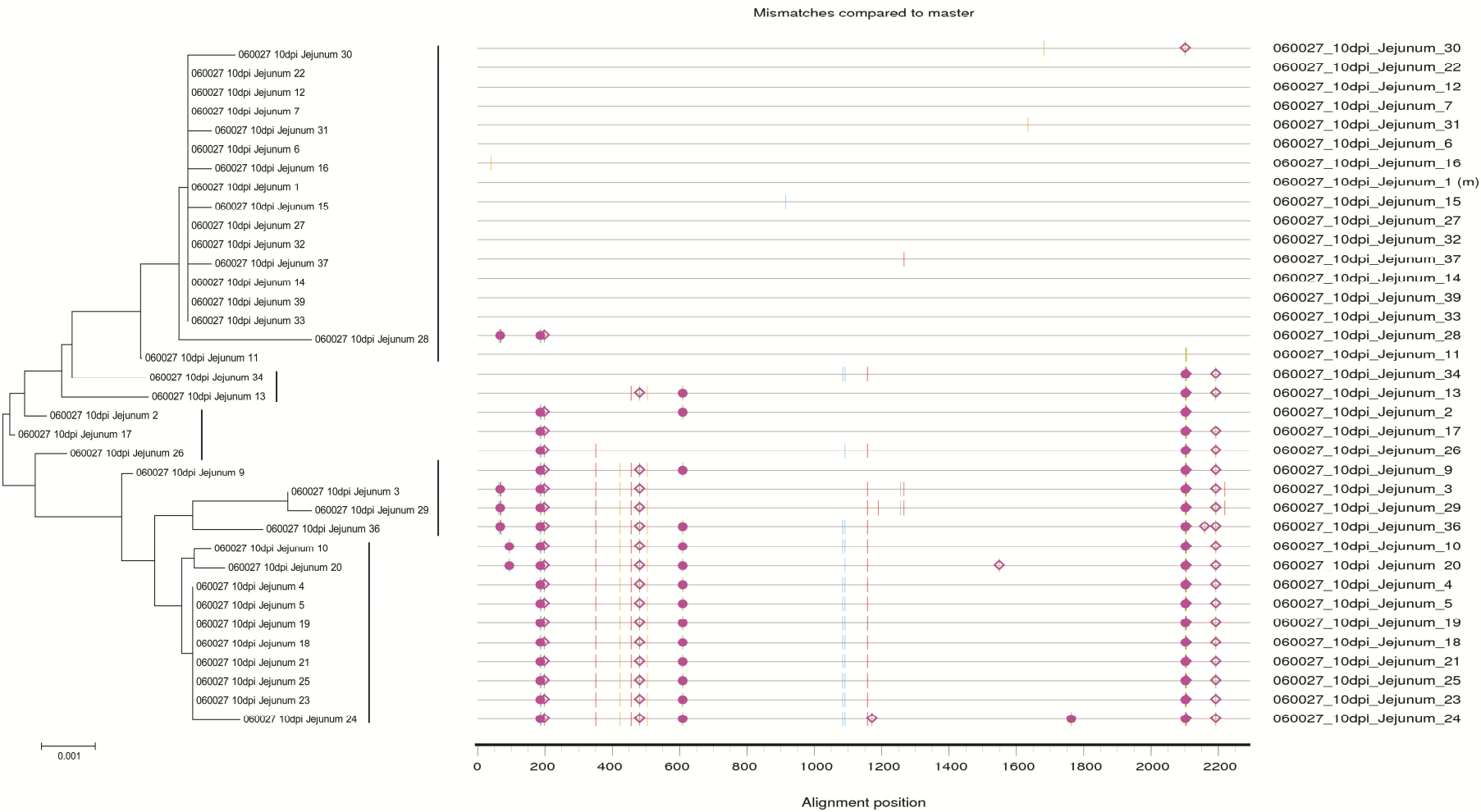

Fig.S5D Rh060027\_Plasma

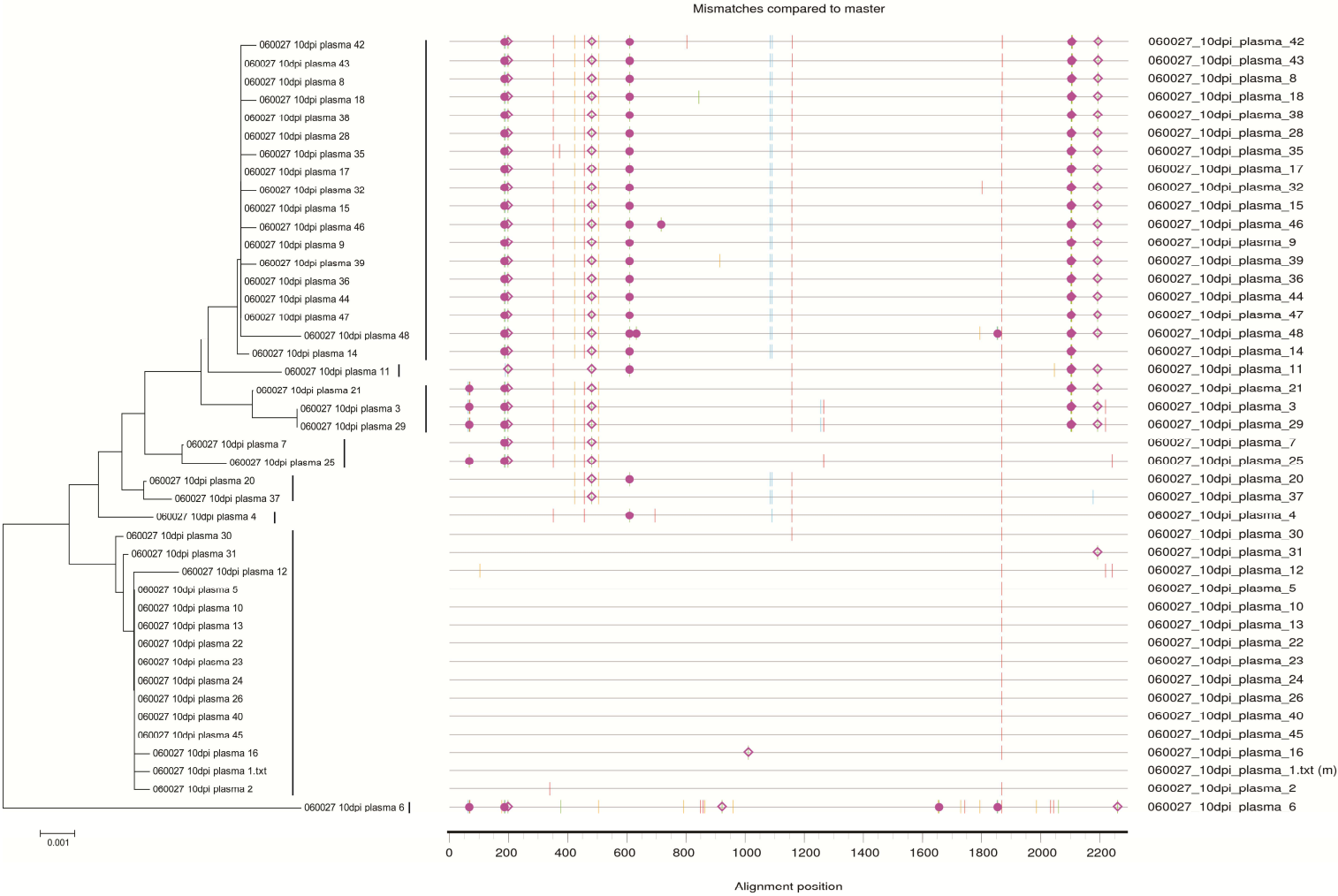

Fig.S5E Rh060027\_Spleen

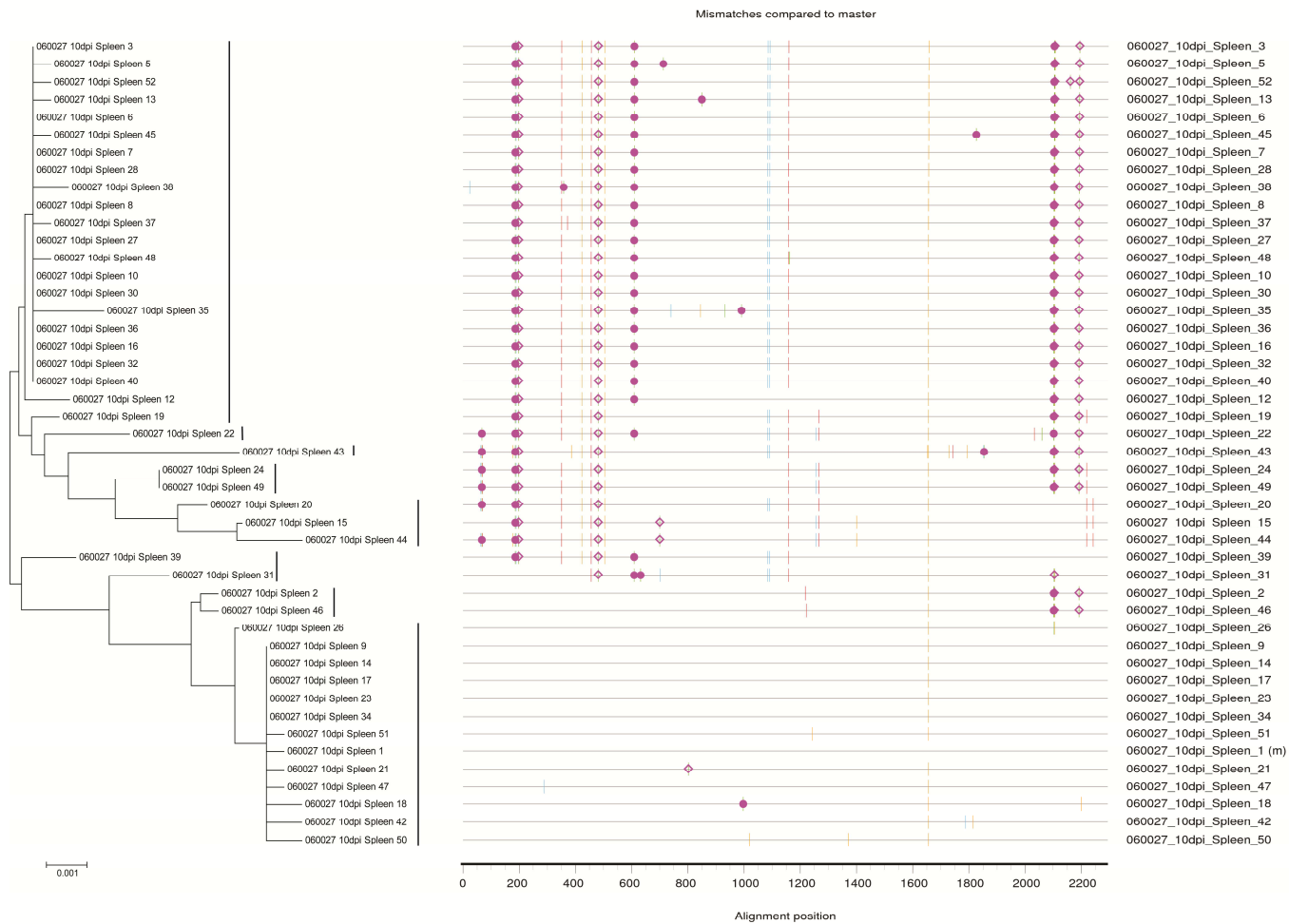

Supplement: Supplementary file 5 [file Image5.PDF]

Fig.S6A Rh060319\_Rectum

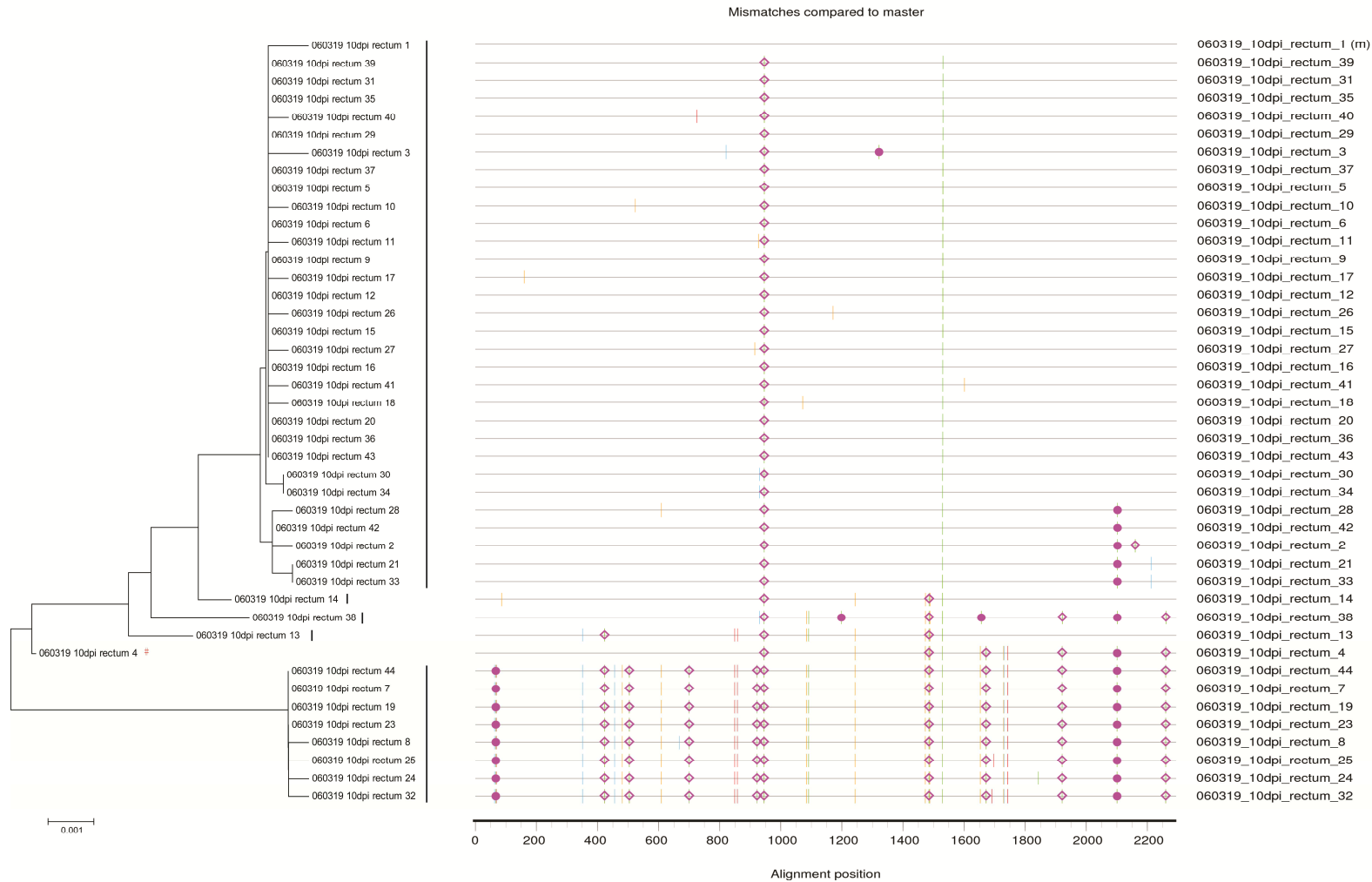

Fig.S6B Rh060319\_Descending colon

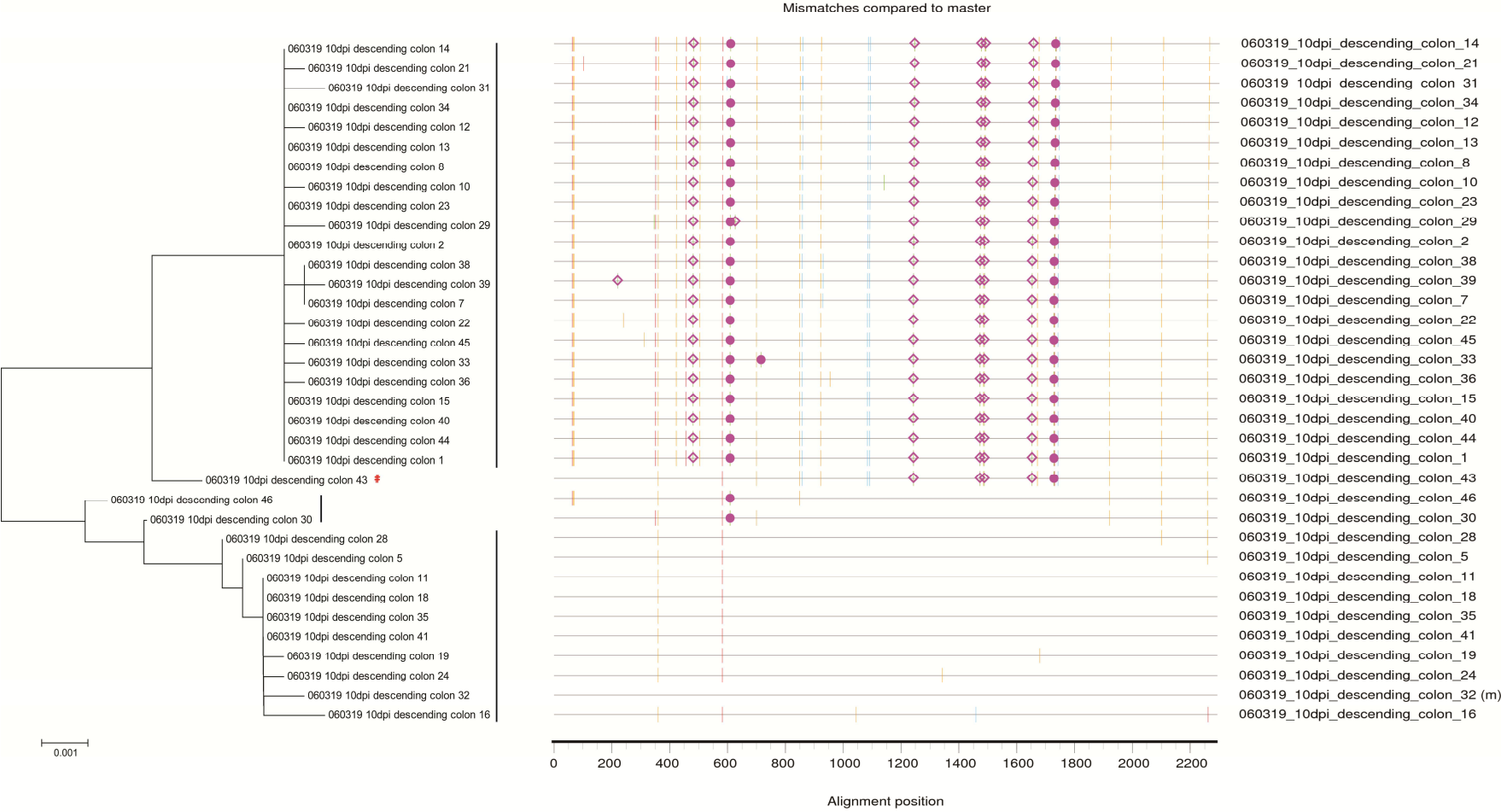

Fig.S6C Rh060319\_Jejunum

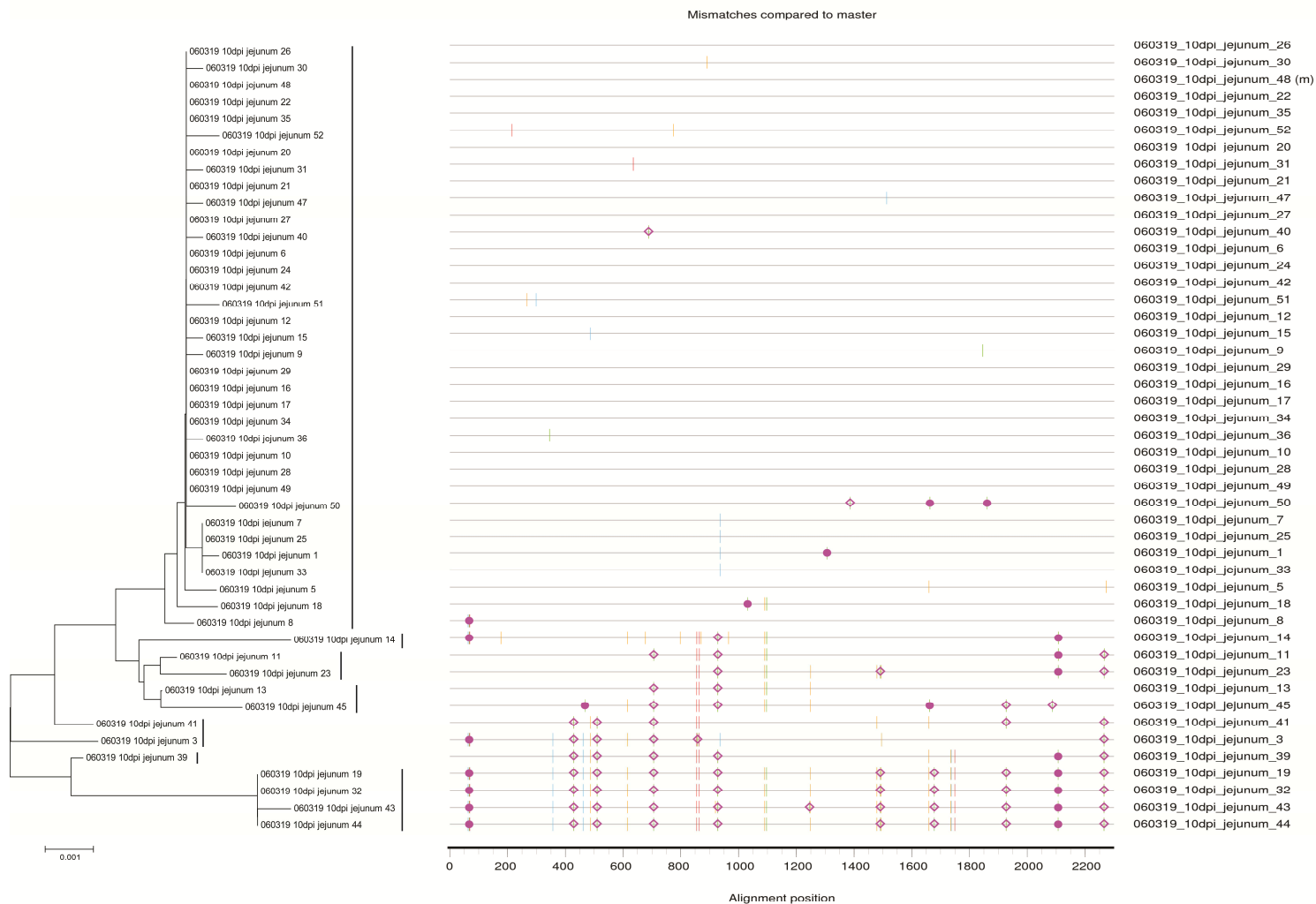

Fig.S6D Rh060319\_Plasma

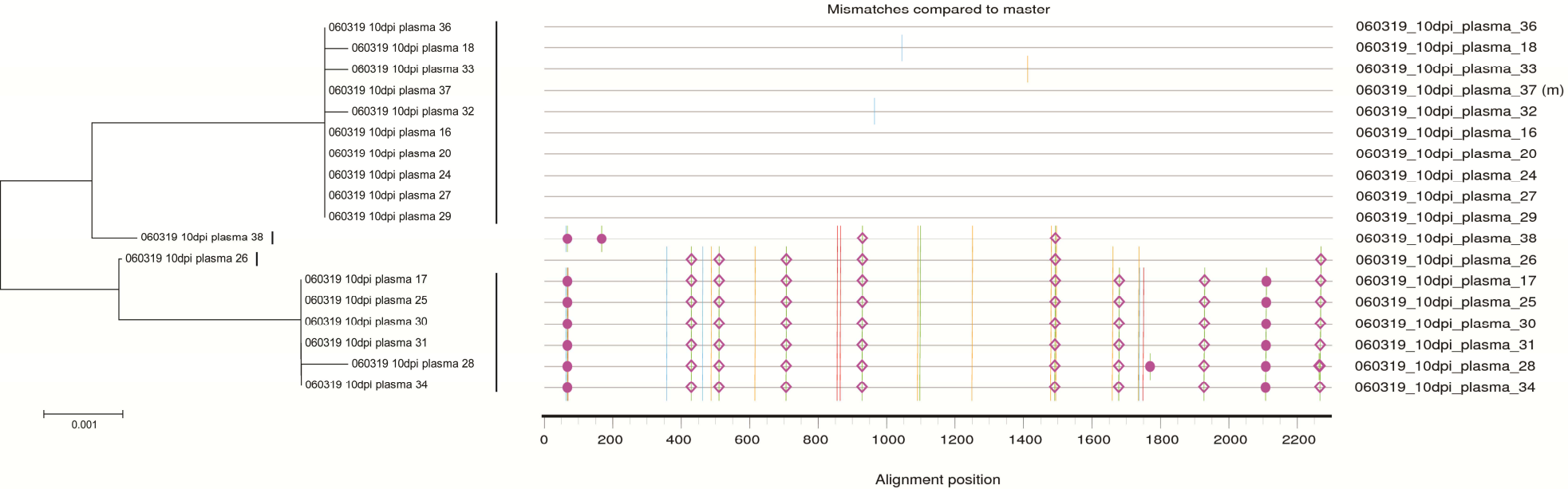

Fig.S6E Rh060319\_Spleen

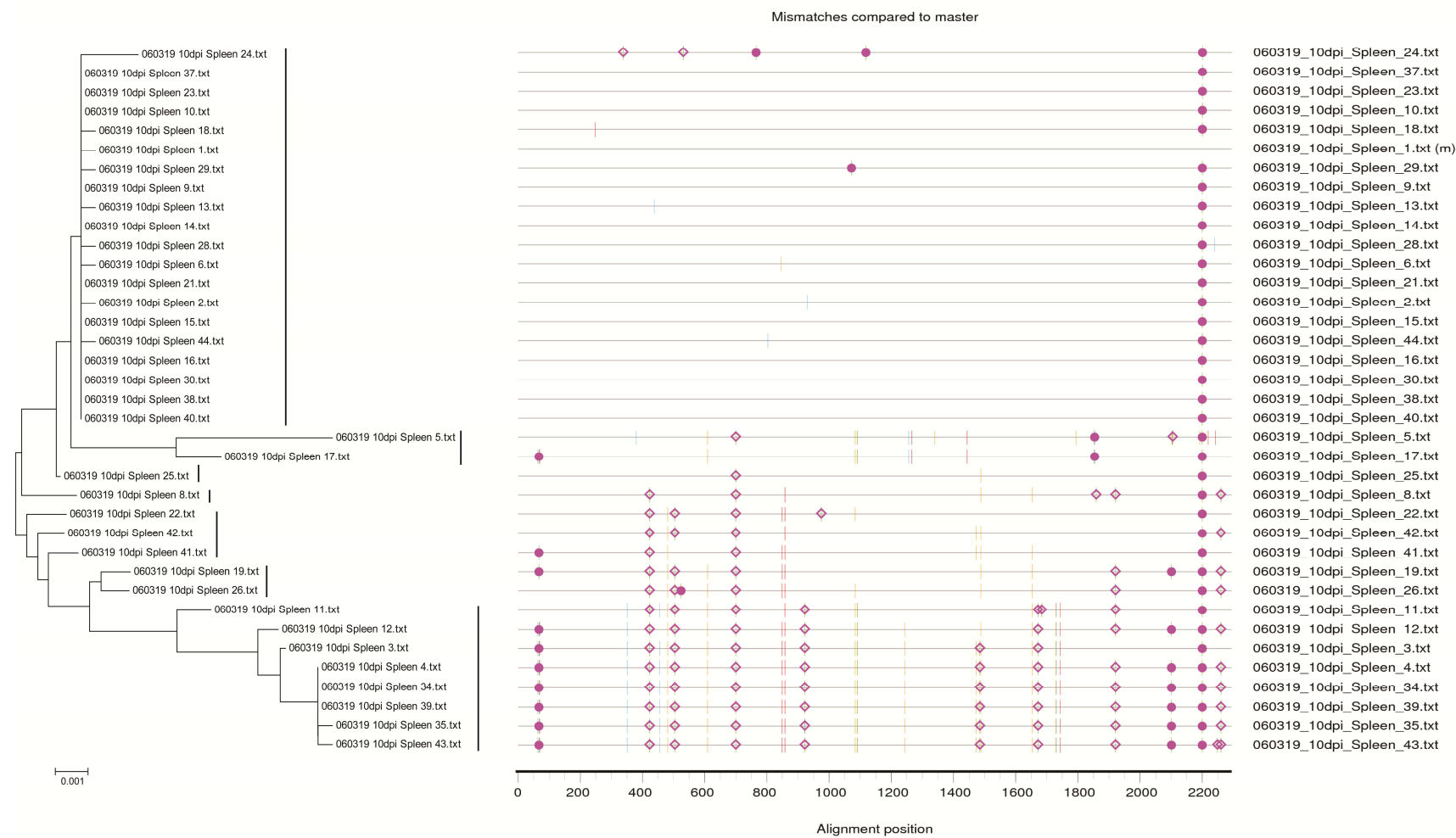

Supplement: Supplementary file 6 [file Image6.PDF]

Fig.S7A

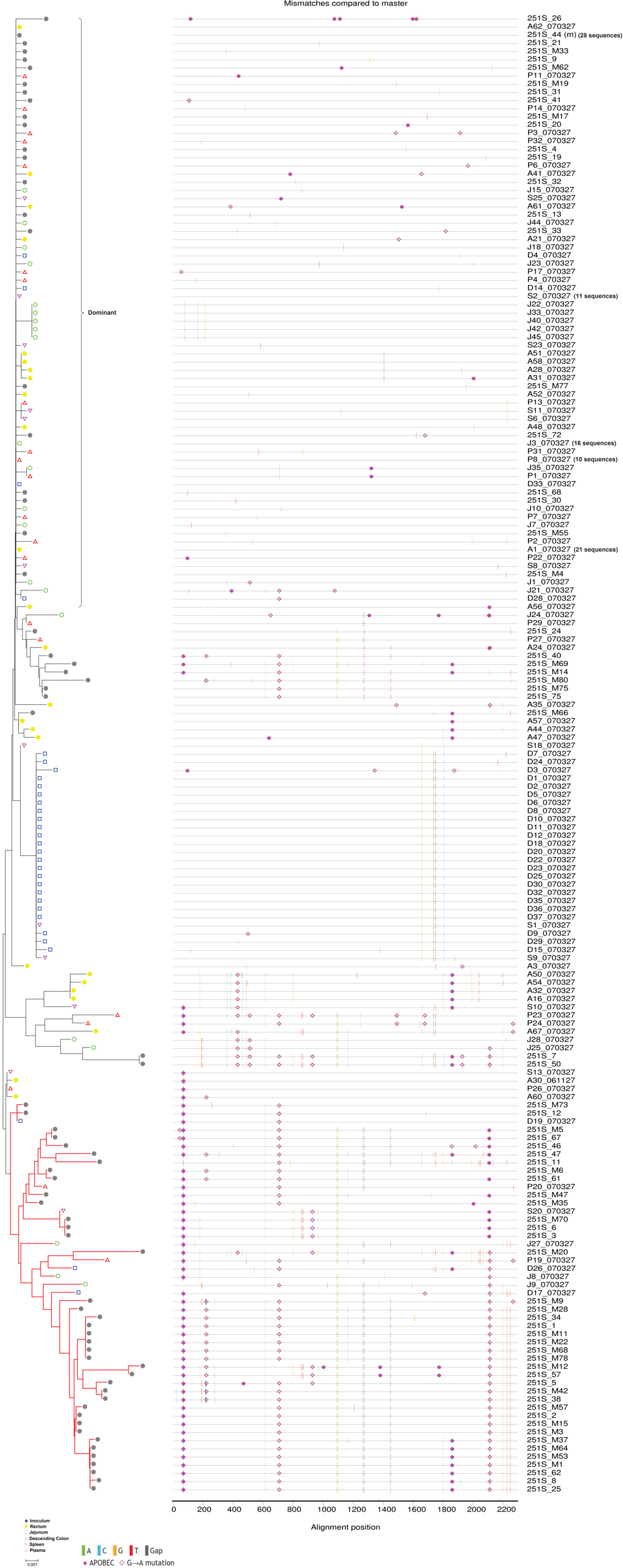

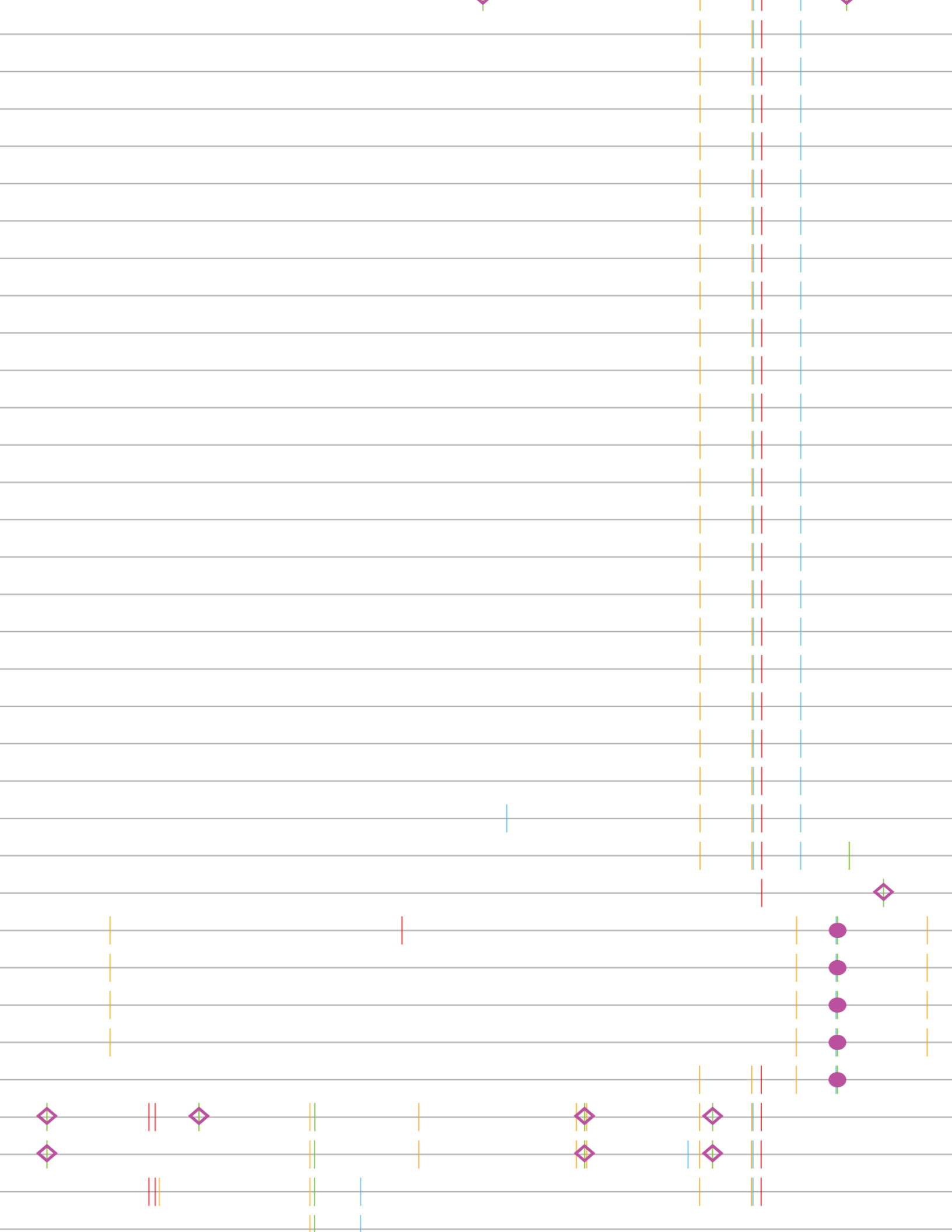

Fig.S7B

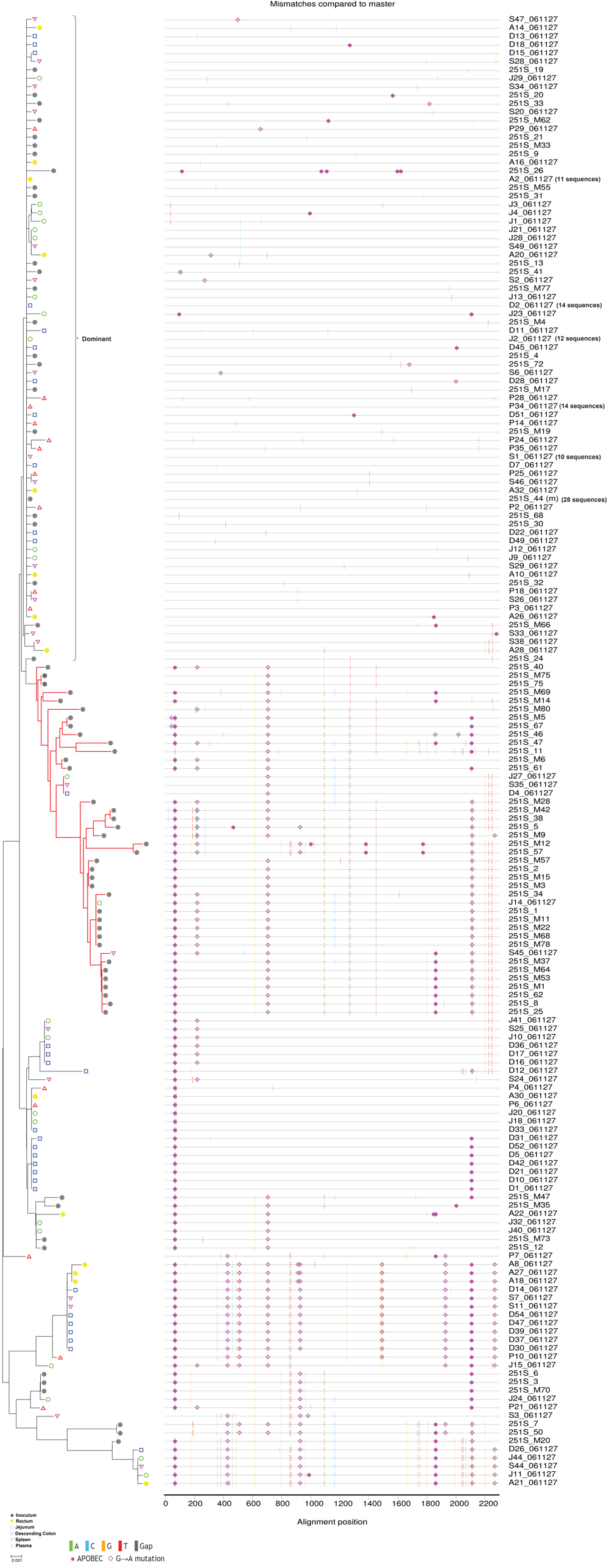

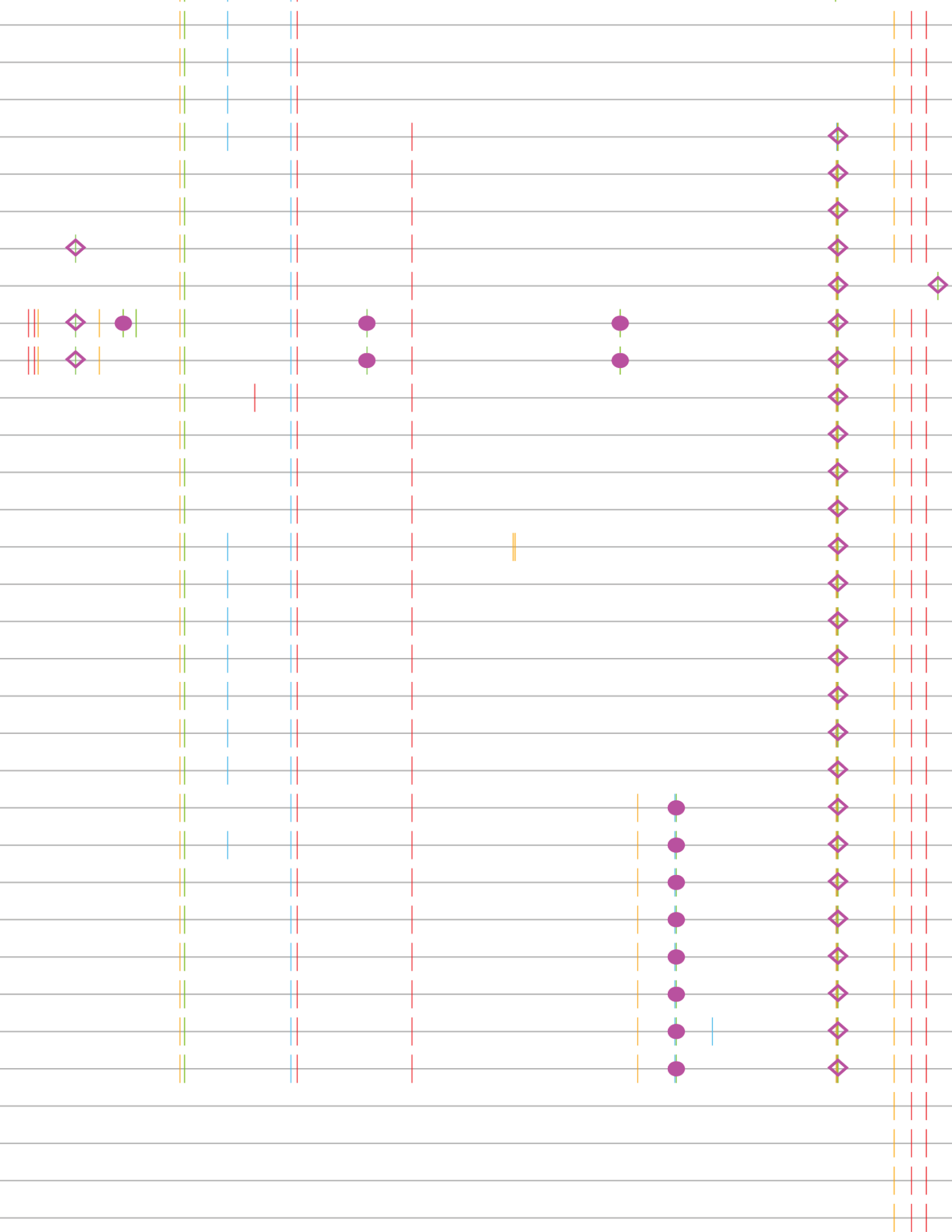

Supplement: Supplementary file 7 [file Image7.PDF]

Fig.S8A

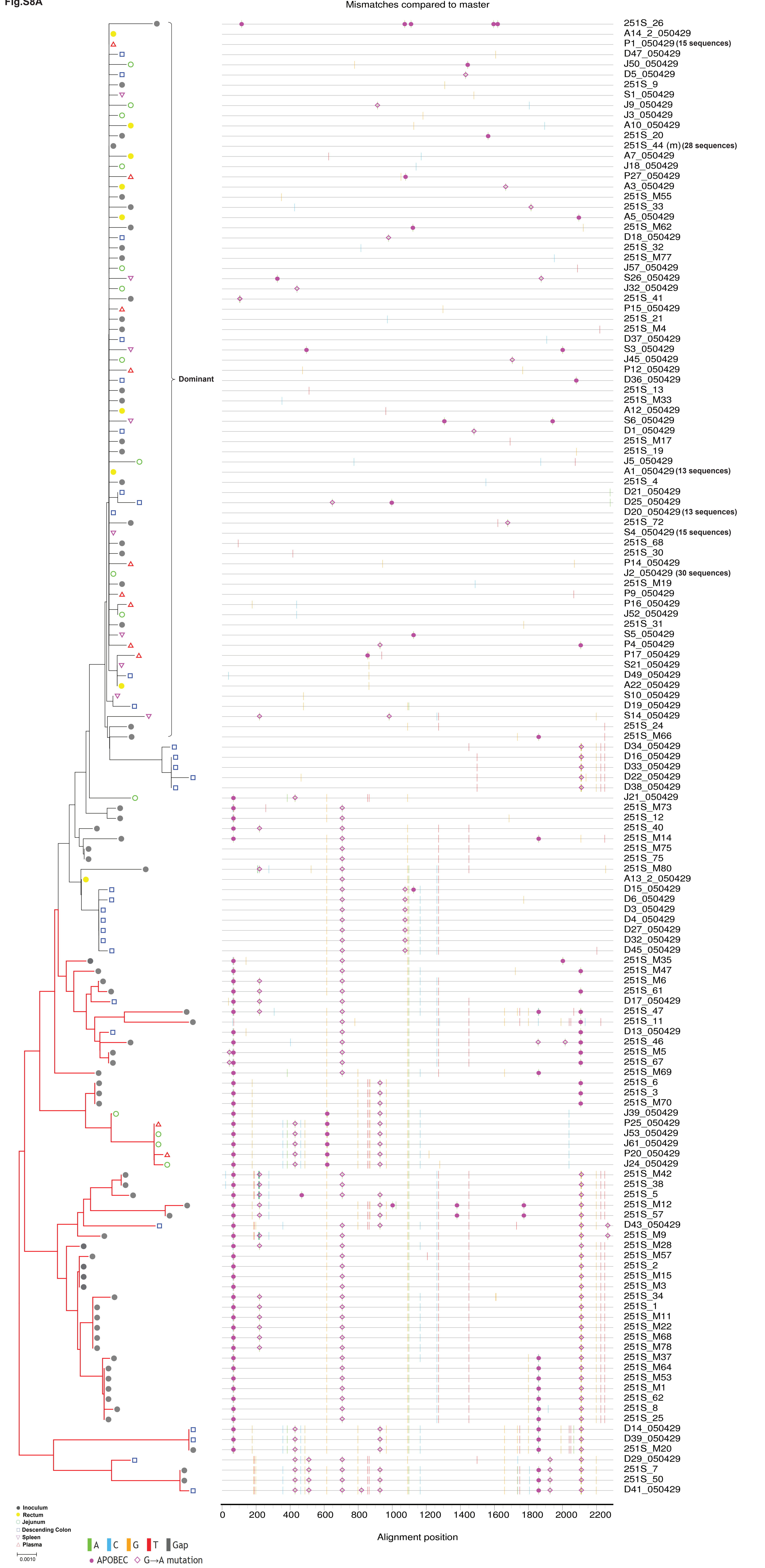

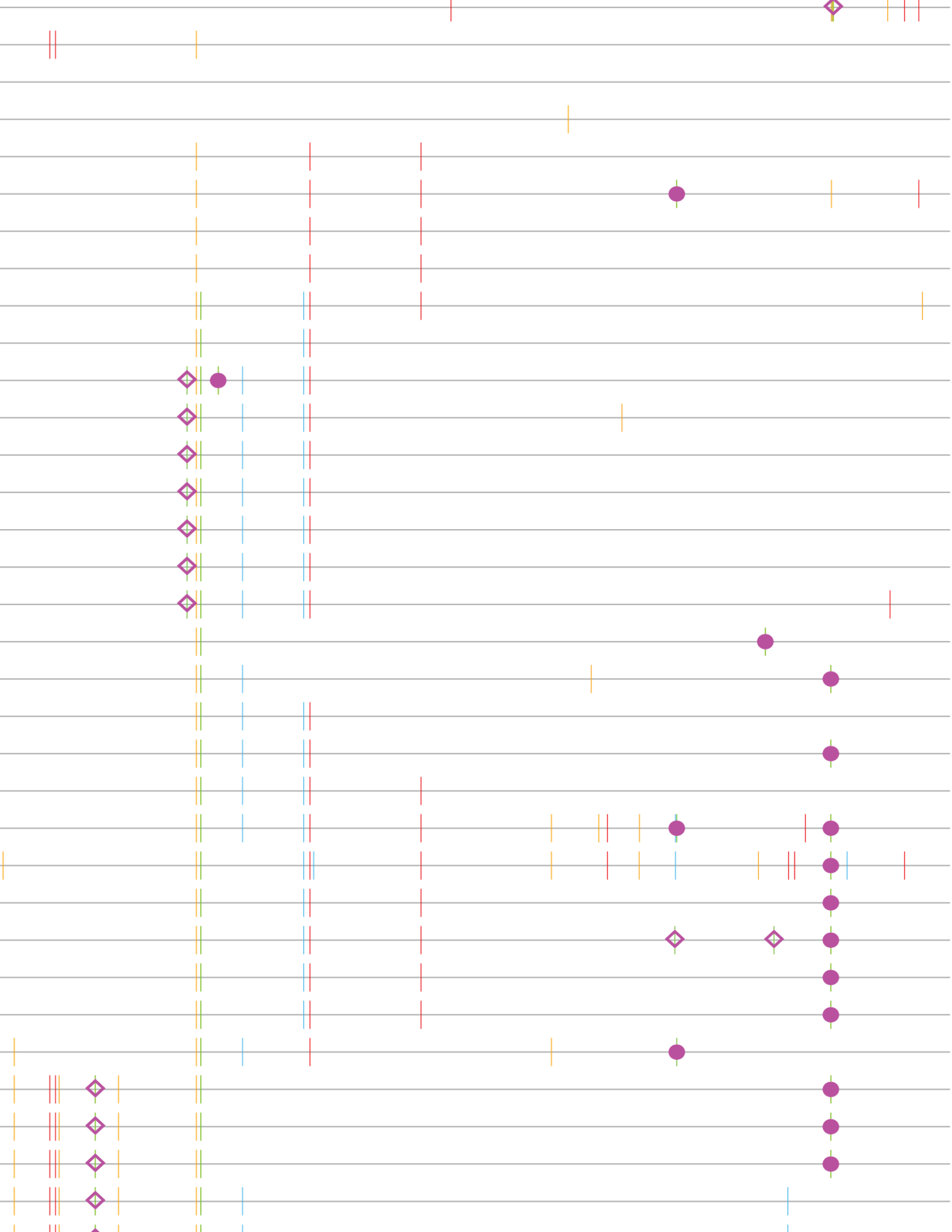



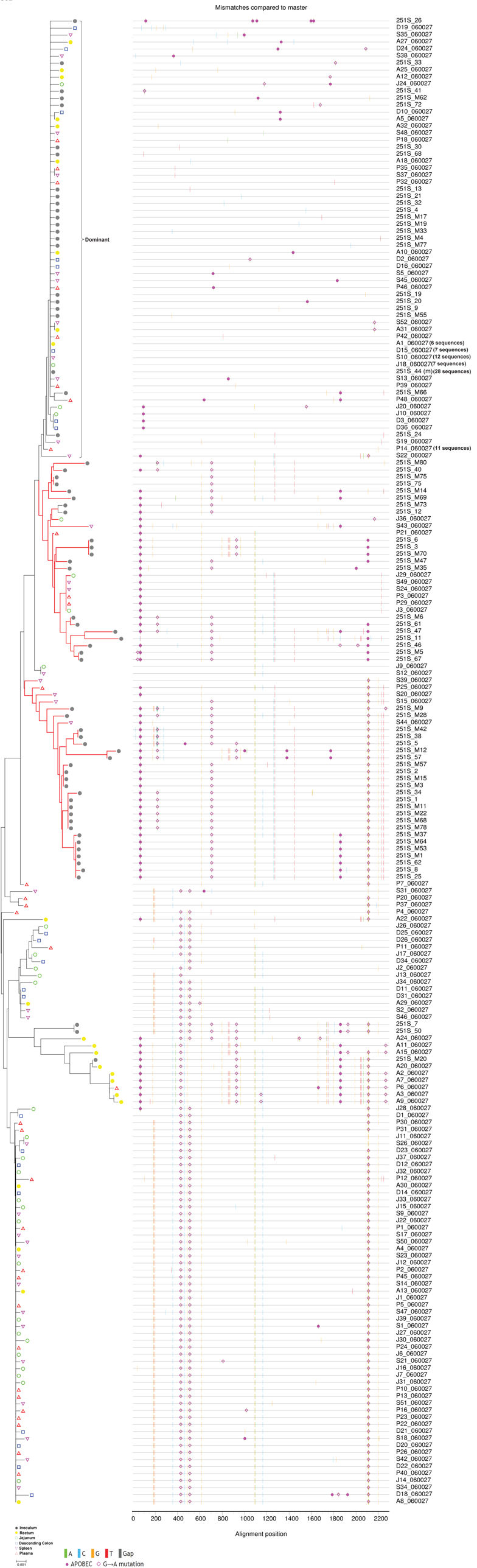

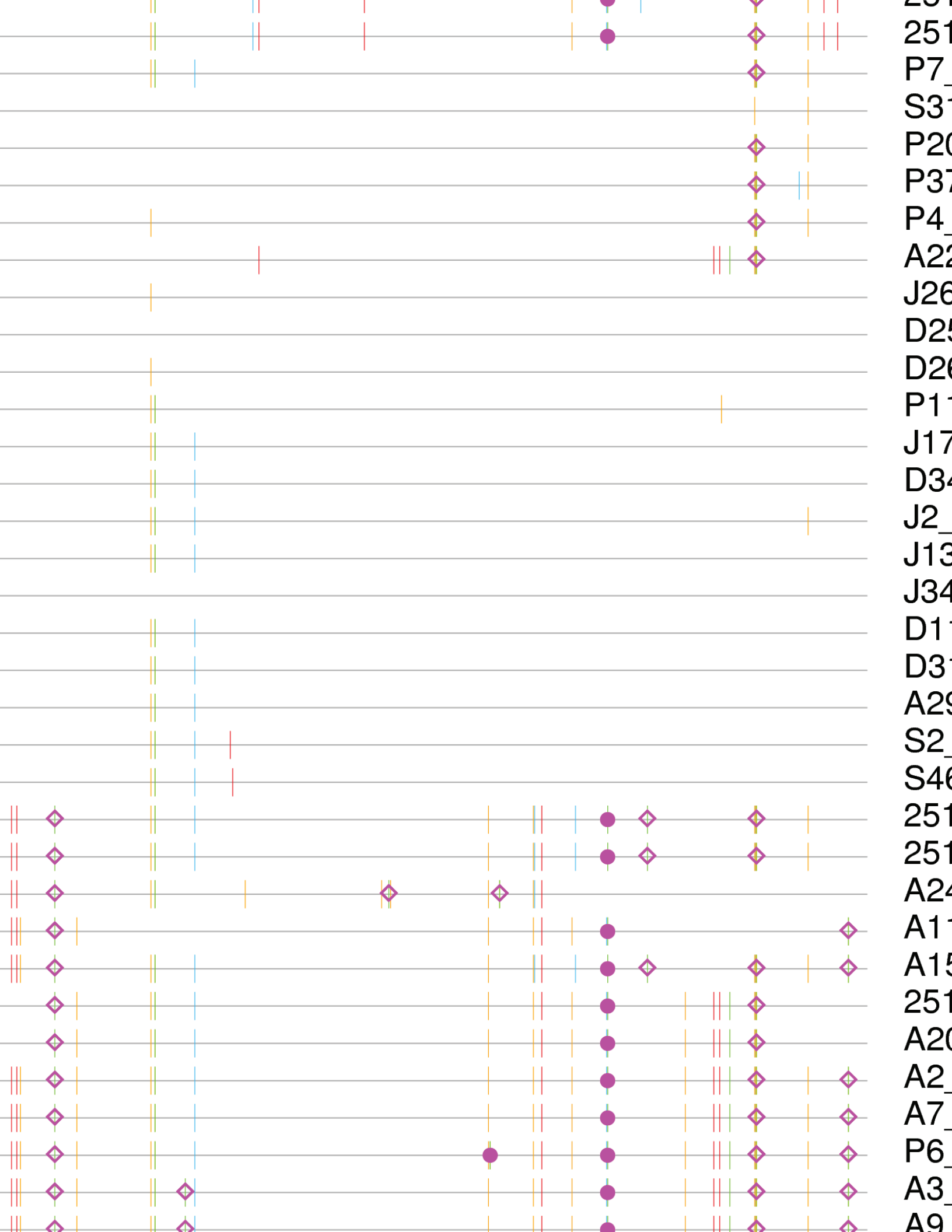

Supplement: Supplementary file 8 [file Image8.PDF]

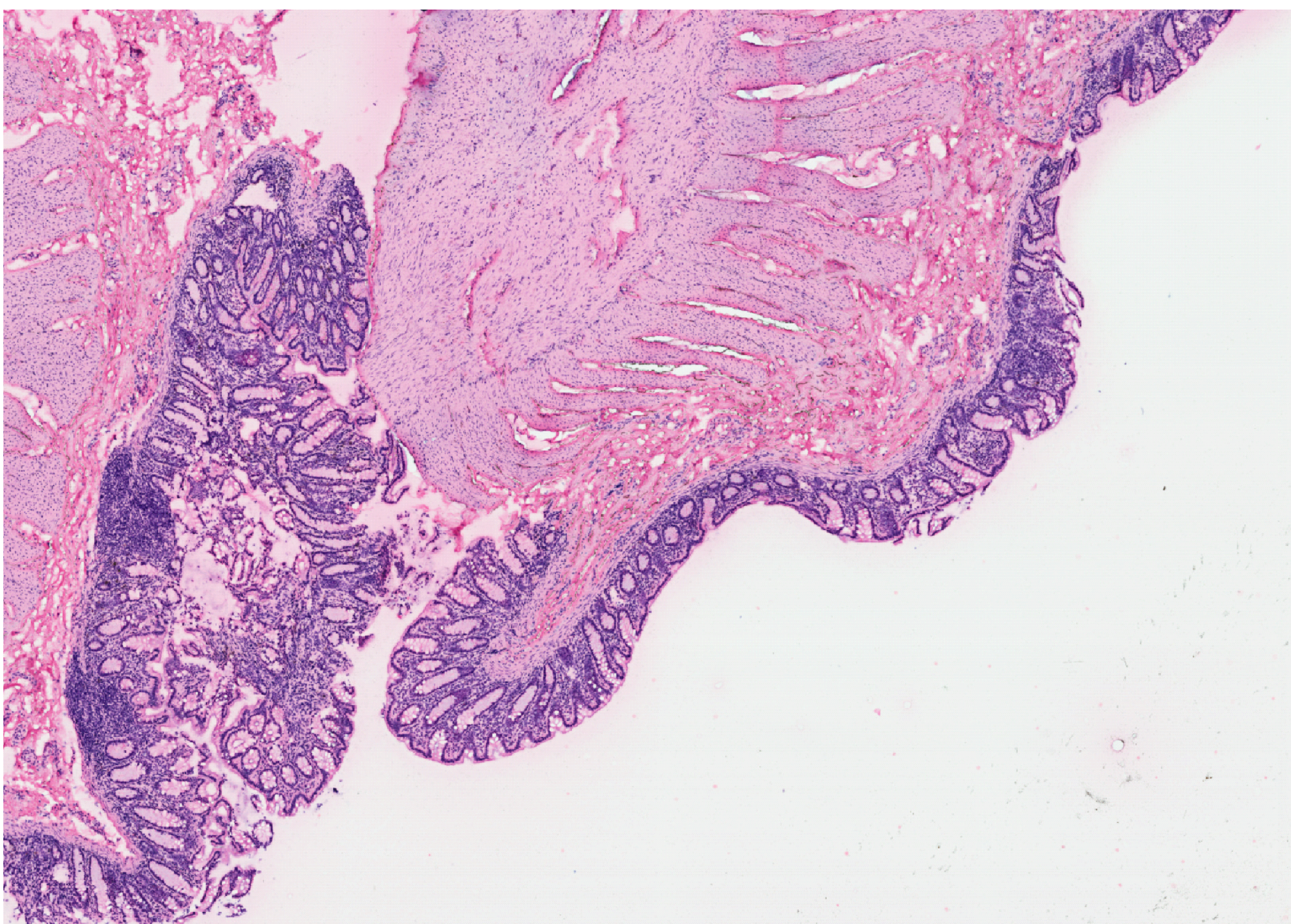

Supplement: Supplementary file 9 [file Image9.PDF]
