# Supplementary material for: Identification of Unequally Represented Founder Viruses Among Tissues in Very Early SIV Rectal Transmission
Source: Front Microbiol. 2018 Mar 29;9:557. doi: 10.3389/fmicb.2018.00557 (PMC5884942; doi:10.3389/fmicb.2018.00557)
Supplement: Supplementary file 10 [file Table1.docx]

**Table S1** The less efficiently transmitted clusters from each monkey phylogenetically related to the same bunch of inoculating virus variants

| **Rhesus macaque ID** | **50429** | **60027** | **60319** | **70327** | **70419** | **61127** |
| --- | --- | --- | --- | --- | --- | --- |
| **Inoculum sequence ID** | 251S_1 | 251S_1 | 251S_1 | 251S_1 | 251S_1 | 251S_1 |
|  | 251S_2 | 251S_2 | 251S_2 | 251S_2 | 251S_2 | 251S_2 |
|  | 251S_3 | 251S_3 | 251S_3 | 251S_3 | 251S_3 |  |
|  | 251S_5 | 251S_5 | 251S_5 | 251S_5 | 251S_5 | 251S_5 |
|  | 251S_6 | 251S_6 | 251S_6 | 251S_6 | 251S_6 |  |
|  | 251S_7 |  |  |  |  |  |
|  | 251S_8 | 251S_8 | 251S_8 | 251S_8 | 251S_8 | 251S_8 |
|  | 251S_11 | 251S_11 | 251S_11 | 251S_11 | 251S_11 | 251S_11 |
|  |  | 251S_12 | 251S_12 | 251S_12 | 251S_12 |  |
|  |  |  | 251S_24 |  | 251S_24 |  |
|  | 251S_25 | 251S_25 | 251S_25 | 251S_25 | 251S_25 | 251S_25 |
|  | 251S_34 | 251S_34 | 251S_34 | 251S_34 | 251S_34 | 251S_34 |
|  | 251S_38 | 251S_38 | 251S_38 | 251S_38 | 251S_38 | 251S_38 |
|  |  | 251S_40 | 251S_40 |  | 251S_40 | 251S_40 |
|  | 251S_46 | 251S_46 | 251S_46 | 251S_46 | 251S_46 | 251S_46 |
|  | 251S_47 | 251S_47 | 251S_47 | 251S_47 | 251S_47 | 251S_47 |
|  | 251S_50 |  |  |  |  |  |
|  | 251S_57 | 251S_57 | 251S_57 | 251S_57 | 251S_57 | 251S_57 |
|  | 251S_61 | 251S_61 | 251S_61 | 251S_61 | 251S_61 | 251S_61 |
|  | 251S_62 | 251S_62 | 251S_62 | 251S_62 | 251S_62 | 251S_62 |
|  | 251S_67 | 251S_67 | 251S_67 | 251S_67 | 251S_67 | 251S_67 |
|  |  | 251S_75 | 251S_75 |  | 251S_75 | 251S_75 |
|  | 251S_M1 | 251S_M1 | 251S_M1 | 251S_M1 | 251S_M1 | 251S_M1 |
|  | 251S_M3 | 251S_M3 | 251S_M3 | 251S_M3 | 251S_M3 | 251S_M3 |
|  | 251S_M5 | 251S_M5 | 251S_M5 | 251S_M5 | 251S_M5 | 251S_M5 |
|  | 251S_M6 | 251S_M6 | 251S_M6 | 251S_M6 | 251S_M6 | 251S_M6 |
|  | 251S_M9 | 251S_M9 | 251S_M9 | 251S_M9 | 251S_M9 | 251S_M9 |
|  | 251S_M11 | 251S_M11 | 251S_M11 | 251S_M11 | 251S_M11 | 251S_M11 |
|  | 251S_M12 | 251S_M12 | 251S_M12 | 251S_M12 | 251S_M12 | 251S_M12 |
|  |  | 251S_M14 | 251S_M14 |  | 251S_M14 | 251S_M14 |
|  | 251S_M15 | 251S_M15 | 251S_M15 | 251S_M15 | 251S_M15 | 251S_M15 |
|  | 251S_M20 |  |  | 251S_M20 |  |  |
|  | 251S_M22 | 251S_M22 | 251S_M22 | 251S_M22 | 251S_M22 | 251S_M22 |
|  | 251S_M28 | 251S_M28 | 251S_M28 | 251S_M28 | 251S_M28 | 251S_M28 |
|  | 251S_M35 | 251S_M35 | 251S_M35 | 251S_M35 | 251S_M35 |  |
|  | 251S_M37 | 251S_M37 | 251S_M37 | 251S_M37 | 251S_M37 | 251S_M37 |
|  | 251S_M42 | 251S_M42 | 251S_M42 | 251S_M42 | 251S_M42 | 251S_M42 |
|  | 251S_M47 | 251S_M47 | 251S_M47 | 251S_M47 | 251S_M47 |  |
|  | 251S_M53 | 251S_M53 | 251S_M53 | 251S_M53 | 251S_M53 | 251S_M53 |
|  | 251S_M57 | 251S_M57 | 251S_M57 | 251S_M57 | 251S_M57 | 251S_M57 |
|  | 251S_M64 | 251S_M64 | 251S_M64 | 251S_M64 | 251S_M64 | 251S_M64 |
|  | 251S_M68 | 251S_M68 | 251S_M68 | 251S_M68 | 251S_M68 | 251S_M68 |
|  | 251S_M69 | 251S_M69 | 251S_M69 |  | 251S_M69 | 251S_M69 |
|  | 251S_M70 | 251S_M70 | 251S_M70 | 251S_M70 | 251S_M70 |  |
|  |  | 251S_M73 | 251S_M73 | 251S_M73 | 251S_M73 |  |
|  |  | 251S_M75 | 251S_M75 |  | 251S_M75 | 251S_M75 |
|  | 251S_M78 | 251S_M78 | 251S_M78 | 251S_M78 | 251S_M78 | 251S_M78 |
|  |  | 251S_M80 | 251S_M80 |  | 251S_M80 | 251S_M80 |

Note: Variants shared by all 6 infected monkey were highlighted in red, shared by 5 monkeys in orange and shared by 4 monkeys in yellow.
