# Supplementary material for: Identification of Unequally Represented Founder Viruses Among Tissues in Very Early SIV Rectal Transmission
Source: Front Microbiol. 2018 Mar 29;9:557. doi: 10.3389/fmicb.2018.00557 (PMC5884942; doi:10.3389/fmicb.2018.00557)
Supplement: Supplementary file 11 [file DataSheet1.doc]

**Supplementary figure S1-S6. Number of T/F viruses in various tissues of each individual monkey.** Transmitted/founder variant clusters were counted by visual inspection of the NJ tree and highlighter diagram. Nucleotide polymorphisms in the highlighter plots are indicated by a colored mark. Thymine is represented in red, guanine in orange, adenine in green, cytosine in blue, filled circles denote APOBEC signatures, open diamonds represent G-to-A conversions, and gaps are shown by gray in the highlighter plots. Bar length indicates 0.001 nucleotide substitutions per site. **S1**, Rh060027; **S2**, Rh070327; **S3**, Rh 070419; **S4**, Rh050429; **S5**, Rh060027; **S6**, Rh060319. Two recombinant T/F variants were identified in S6A and S6B, respectively (marked with #).

**Supplementary figure S7-S8. N-J tree and Highlighter plots of SGA-derived *env* nucleotide sequences from 6 dpi macaques Rh070327, Rh061127 and 10 dpi macaques Rh060027, Rh050429.** Sequences derived from different tissue compartments are shown in N-J phylogeny and Highlighter plots. In the phylogeny plots, the inoculum viruses are depicted in closed grey circles, rectum viruses in closed yellow circles, jejunum viruses in green circles, descending colon viruses in blue squares, spleen viruses in purple downward triangles, and peripheral blood viruses in red upward triangles. The red branches highlight a cluster of variants that was absent from rectum. The right brackets indicate the dominant variants. Identical sequences in the dominant cluster are represented by only one sequence for each tissue compartment. Actual number of sequences is shown in the bracket following the seq ID. Bar length represents 0.001 nucleotide substitutions per site. **S7A**, Rh070327; **S7B**, Rh061127; **S8A**, Rh050429; **S8B**, Rh060027.

**Supplementary figure S9 In situ hybridization assay for the rectum tissue of Rh060327 (non-infected control).** This sample was tested as a negative control for the ISH assay.
